# Biocompatible Ti_3_Au–Ag/Cu thin film coatings with enhanced mechanical and antimicrobial functionality

**DOI:** 10.1186/s40824-023-00435-1

**Published:** 2023-09-25

**Authors:** Cecil Cherian Lukose, Ioannis Anestopoulos, Iraklis-Stavros Panagiotidis, Guillaume Zoppi, Anna M. Black, Lynn G. Dover, Leon Bowen, Ángel Serrano-Aroca, Terence Xiaoteng Liu, Lorenzo Mendola, Davide Morrone, Mihalis I. Panayiotidis, Martin Birkett

**Affiliations:** 1 Department of Mechanical and Construction Engineering, Northumbria University, Newcastle Upon Tyne, NE1 8ST UK; 2 Department of Cancer Genetics, Therapeutics and Ultrastructural Pathology, The Cyprus Institute of Neurology and Genetics, 1683 Nicosia, Cyprus; 3 Department of Mathematics, Physics and Electrical Engineering, Northumbria University, Newcastle Upon Tyne, NE1 8ST UK; 4 Department of Applied Sciences, Northumbria University, Newcastle Upon Tyne, NE1 8ST UK; 5 Department of Physics, G.J. Russell Microscopy Facility, Durham University, Durham, DH1 3LE UK; 6 Biomaterials and Bioengineering Lab, Centro de Investigación Traslacional San Alberto Magno, Universidad Católica de Valencia San Vicente Mártir, C/Guillem de Castro 94, 46001 Valencia, Spain; 7 Nanovea Inc., 6 Morgan Ste 156, Irvine, CA 92618 USA

**Keywords:** Ti_3_Au, Super hard coating, Biocompatible, Antimicrobial, Controlled doping

## Abstract

**Background:**

Biofilm formation on medical device surfaces is a persistent problem that shelters bacteria and encourages infections and implant rejection. One promising approach to tackle this problem is to coat the medical device with an antimicrobial material. In this work, for the first time, we impart antimicrobial functionality to Ti_3_Au intermetallic alloy thin film coatings, while maintaining their superior mechanical hardness and biocompatibility.

**Methods:**

A mosaic Ti sputtering target is developed to dope controlled amounts of antimicrobial elements of Ag and Cu into a Ti_3_Au coating matrix by precise control of individual target power levels. The resulting Ti_3_Au-Ag/Cu thin film coatings are then systematically characterised for their structural, chemical, morphological, mechanical, corrosion, biocompatibility-cytotoxicity and antimicrobial properties.

**Results:**

X-ray diffraction patterns reveal the formation of a super hard β-Ti_3_Au phase, but the thin films undergo a transition in crystal orientation from (200) to (211) with increasing Ag concentration, whereas introduction of Cu brings no observable changes in crystal orientation. Scanning and transmission electron microscopy analysis show the polyhedral shape of the Ti_3_Au crystal but agglomeration of Ag particles between crystal grains begins at 1.2 at% Ag and develops into large granules with increasing Ag concentration up to 4.1 at%. The smallest doping concentration of 0.2 at% Ag raises the hardness of the thin film to 14.7 GPa, a 360% improvement compared to the ∼4 GPa hardness of the standard Ti_6_Al_4_V base alloy. On the other hand, addition of Cu brings a 315—330% improvement in mechanical hardness of films throughout the entire concentration range of 0.5—7.1 at%. The thin films also show good electrochemical corrosion resistance and a > tenfold reduction in wear rate compared to Ti_6_Al_4_V alloy. All thin film samples exhibit very safe cytotoxic profiles towards L929 mouse fibroblast cells when analysed with Alamar blue assay, with ion leaching concentrations lower than 0.2 ppm for Ag and 0.08 ppm for Cu and conductivity tests reveal the positive effect of increased conductivity on myogenic differentiation. Antimicrobial tests show a drastic reduction in microbial survival over a short test period of < 20 min for Ti_3_Au films doped with Ag or Cu concentrations as low as 0.2—0.5 at%.

**Conclusion:**

Therefore, according to these results, this work presents a new antimicrobial Ti_3_Au-Ag/Cu coating material with excellent mechanical performance with the potential to develop wear resistant medical implant devices with resistance to biofilm formation and bacterial infection.

**Graphical Abstract:**

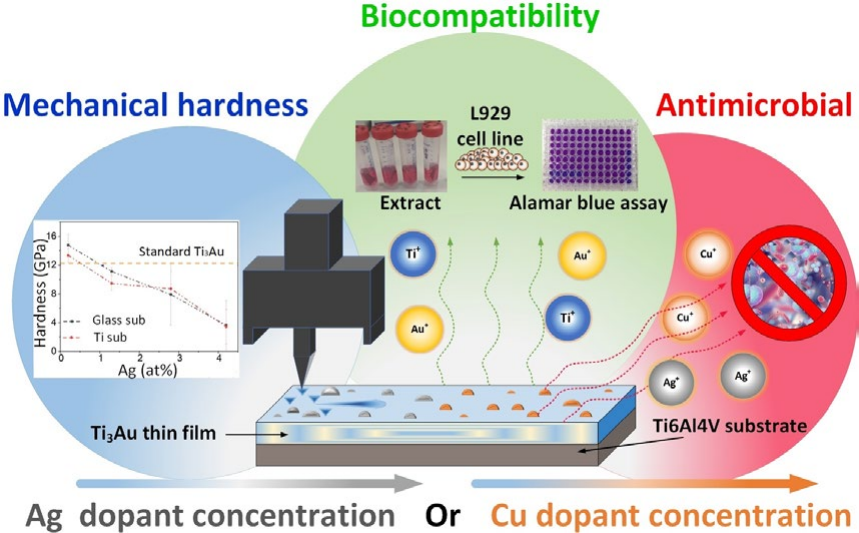

**Supplementary Information:**

The online version contains supplementary material available at 10.1186/s40824-023-00435-1.

## Background

Interest in intermetallic alloys of titanium (Ti) and gold (Au), especially Ti_3_Au, is increasing due to its excellent biocompatibility combined with its enhanced hardness value of > 12 GPa [1–5]. Ti-based alloys are widely used in aerospace and military applications requiring high strength-to-weight ratio, while medical grade Ti alloys like Ti_6_Al_4_V (or Ti64) are of great importance for the medical implant device sector because of their excellent mechanical strength, superior osteointegration, low ion formation and excellent corrosion resistance. While Ti_6_Al_4_V is an established material for artificial joint manufacture, its comparatively lower mechanical hardness and health concerns over leaching of Al and V ions [6–8], has given way to other ceramic-based materials like oxidised-zirconium, which is produced by gradual oxidation of pure zirconium, resulting in superior surface hardness values > 12 GPa, making it an industry favourite for developing long-life load-bearing artificial joints [9, 10]. However, recent studies have shown that the wear rate of articulating surfaces of artificial implants like knee, hip, elbow or shoulder joints is accelerated when the harder oxide surface layer wears off and exposes the softer base metal [9–12].

Ti and Au elements are well known for their biocompatibility and corrosion resistance, which makes them good choices for various implant applications. Initial studies on TiAu, like that of Oh et al*.* [13], focussed on the excellent biocompatibility and improvement in corrosion resistance achieved by limited Au addition (0–20 at%) into a Ti matrix in bulk format and a substantial improvement in mechanical hardness from ~ 2 to 3.5 GPa compared to commercially pure Ti samples, was also observed with heat treatment, caused by an α to β phase transition of the Ti matrix. A better understanding of the Ti-Au interaction was then developed by Martinez et al*.* [14], and the first principle studies from Rajagopalan et al*.* [15], revealed greater insight to predict structural, mechanical and thermal properties of the Ti_3_Au intermetallic. Later, Lee et al*.* [16], associated increased hardness of Ti-Au alloys (Au concentration 0–40 at%) to precipitate hardening caused by evolution of the Ti_3_Au intermetallic phase from an Au concentration of 15.3 at% onwards. However this work failed to identify different phases of Ti_3_Au, which is now known to have a serious effect on the outcome of mechanical hardness, thanks to the ground breaking work of Svanidze et al*.* in 2016 [2], on arc melting Ti-Au in varying compositions. Svanidze et al*.* found that the mechanical hardness of the Ti-Au alloy increased non-monotonously, reaching a peak hardness of ~ 7.85 GPa when the intermetallic alloy forms a cubic Ti_3_Au compound [2, 3]. The mechanical performance of the Ti_3_Au intermetallic was observed to outperform many other commonly used medical alloy systems in terms of hardness, like stainless steel, cobalt chrome and Ti_6_Al_4_V alloys [2, 17]. This enhancement in hardness was understood to be caused by the higher mass density of Au which allows it to exhibit higher valence electron density, leading to increased bond length and higher hardness for the final alloy. It was observed that even though Ti_3_Au starts forming between 10–35 at% of Au, it can exist in two different α and β forms, depending upon the fabrication conditions and stoichiometric composition. The enhancement in hardness is also caused by a difference in their respective atomic arrangement. Au atoms exist in 12-fold coordination for both α and β phases, whereas Ti atom coordination changes form 12-fold (8 Ti and 4 Au) in the α phase to 14-fold (10 Ti and 4 Au) in the β phase. This results in denser packing of the β phase unit cell, which presents a higher energy barrier for slipping of dislocation planes within the lattice system and therefore higher hardness compared to its α phase. One promising solution to extend the lifetime of artificial joint implants made of Ti_6_Al_4_V alloy is to coat their articulating surfaces with a hard, biocompatible Ti_3_Au thin film which will provide enhanced wear performance while reducing the ion leaching potential of Al and V elements. This could also result in a reduction in implant material cost due to relatively low cost of Ti_6_Al_4_V alloy ($10/kg) when compared to zirconium ($35/kg) [18] used to manufacture oxidised-zirconium implant devices.

Based upon these results for bulk Ti-Au alloys, Karimi et al. [4], explored the mechanical performance of the Ti-Au alloy in thin film format and observed an improvement in ductility due to satisfaction of the Pugh criterion (B/G < 1.75) [4, 15, 19] as the underlying substrate reinforced the integrity of the Ti-Au thin film above it. Recently [1], we showed that excellent mechanical hardness of ~ 12 GPa can be achieved with Ti_3_Au thin films deposited on Ti_6_Al_4_V substrates by carefully controlling the Au concertation in the Ti matrix. Our work also established a strong dependency of crystallization of the Ti_3_Au phase on the substrate temperature, leading to preferential orientation of the β phase of Ti_3_Au with a substrate temperature of 450˚C [1, 16]. Further to this it was also established that reducing the deposition pressure to 0.3 Pa, plays a crucial role in achieving Ti_3_Au thin films with denser columnar microstructure and improved hardness values of > 12.5 GPa.

While superior mechanical hardness and biocompatibility makes Ti_3_Au thin film coatings suitable for load-bearing implant applications, its functionality can be further enhanced by imparting it with antimicrobial properties to help combat implant associated infections (IAI). When a foreign body like an artificial implant is introduced in the body, it provides a substrate for microorganisms to colonize and eventually form a protective biofilm that is impervious to antibiotic treatment and response from the host immune system [20–23], resulting in an IAI. The associated infection often leads to failure of the implant device and a requirement for costly corrective surgery which can have a significant impact on both the patient and healthcare system [21]. One study showed that infection associated implant failures account for 26% of all healthcare associated infections [20], whereas another study describes IAI’s as the most pulverizing post-operative problem associated with the artificial implant installation process [21].

The antimicrobial performance of commercially pure Ti is very poor, and while development of surface oxides like TiO_2_, can improve osteo-inductive properties, the bactericidal effects are still very limited and insufficient to overcome biofilm formation and prevent IAI’s [21, 24–26]. Even established Ti alloys like Ti_6_Al_4_V, Ti-Ni, Ti-Nb, and Ti-Zr lack antimicrobial performance and need special surface treatment like oxygen plasma immersion ion implantation (O-PIII) to observe any bactericidal activity [27]. Very recently, Fu et al*.* [28], reported on a Ti-Au material system with antimicrobial action against the Gram-positive bacteria *S. aureus*. The antibacterial performance was directly proportional to the ratio of Ti_3_Au intermetallic phase and was attributed to development of hot electrons which damage the reactive oxygen species (ROS) homeostasis of bacteria, causing oxidative cell stress and prevention of biofilm formation.

The antimicrobial performance of Ti-based alloys can be further enhanced with the addition of biocidal elements such as silver (Ag) and copper (Cu). Work done by Lie et al*.* [29], shows that a mere 1–4 at% of Ag in a Ti-Ag alloy can achieve a 90–98% reduction in *S. aureus* bacterial colonies, within 24 h of exposure. Another work by Ewald et al*.* [30], showed that 0.7–4 at% of Ag, releasing 0.5–2.3 ppb of Ag + ions over 24 h of incubation time period is sufficient to cause a 32–64% reduction in bacterial colonies of the *Klebsiella pneumoniae* bacteria strain. Dosunmu et al*.* [31], attributed the antibacterial properties of Ag nano particles to degradation of bacterial cell membrane, downregulation of bacterial virulence genes, and their induction of general and oxidative stress, while Gordon et al. [32], showed that Ag ion interaction leads to deactivation of key enzymes like succinate dehydrogenase mediated by their binding to thiol groups and on their induction of hydroxyl radical formation, which results in DNA damage. Similarly for Cu, Ji et al*.* [33], observed that a Cu concentration of 3–10 at% is sufficient to cause a 99% reduction in bacterial colonies of the Escherichia coli (*E. coli*) strain due to the release of high levels of Cu2 + ions (40–100 ppm over 24 h) which leads to destruction of the bacterial cell wall and membrane. Another work from Wu et al*.* [34], associates the excellent antimicrobial properties of a Ti-Cu alloy containing 5 at% Cu, to the formation of acicular Ti_2_Cu intermetallic at higher processing temperature with higher release rate of Cu2 + ions. Work done by Javadhesari et al*.* [35] on Ti-Cu alloys*,* showed a 96% reduction in *E. coli* and *S. aureus* (24 h time period) with Cu ion release levels as low as 6.7–11 ppb, while also achieving a two-fold increase in mechanical hardness to 10 GPa, when compared to the standard Ti_6_Al_4_V alloy. The antimicrobial performance of the Cu containing surface is attributed to the release of Cu2 + ions which enter the bacterial cell membrane and produce intracellular ROSs like superoxide radical anion (O_2_^−^), hydrogen peroxide (H_2_O_2_), and highly reactive hydroxyl radicals (OH). These reactive species interact with biological molecules within bacteria like lipids, proteins and nucleic acid, causing oxidative damage to the organism [35]. Other works also support the possible mechanism behind antimicrobial performance of Cu ions to be the extraction of electrons from bacteria which leads to damaged cell permeability and thereby loss of bacterial cytoplasm and oxidation of nucleus [33]. Studies have shown that such nanoparticle based interventions from implant surfaces can form a sustainable multifunctional barrier against intracellular infections caused by multi drug resistant (MDR) or antimicrobial resistance (AMR) bacterial species and further can also exhibit wound healing properties [36].

It is clear that by developing a biocompatible coating with enhanced hardness and antimicrobial properties, artificial load bearing hip, knee, elbow and shoulder implants with longer lifecycle can be manufactured, which will considerably reduce the risk of IAI’s. Apart from artificial joint implants, such super hard biocompatible alloys can be used to manufacture scratch resistance luxury watches/jewellery, long lasting biocompatible wearable devices, light weight human exoskeleton systems for extreme environments and antimicrobial applications such as high touch surfaces, food packaging and water treatment. In this work, we make the first fundamental attempt to dope the biocompatible β-Ti_3_Au thin film structure with small quantities (< 10 at%) of biocidal Ag and Cu elements to impart strong antimicrobial functionality without affecting the excellent mechanical hardness of this alloy system. We demonstrate new super hard Ti_3_Au biocompatible thin film coatings doped with X (x = Ag, Cu) with enhanced hardness and wear resistance and potential to develop medical implant devices with excellent resistance to biofilm formation and bacterial infection. The novelty of the proposed Ag/Cu doped Ti_3_Au coating lies in its double acting approach to extend the lifetime of artificial implants, firstly by enhancing the wear resistance of implant articulating surfaces and secondly through its strong resistance against biofilm formation to reduce the risk of implant failure due to IAI’s during the critical initial hours post-surgery.

## Methods

### Thin film deposition

**Fig. 1:**
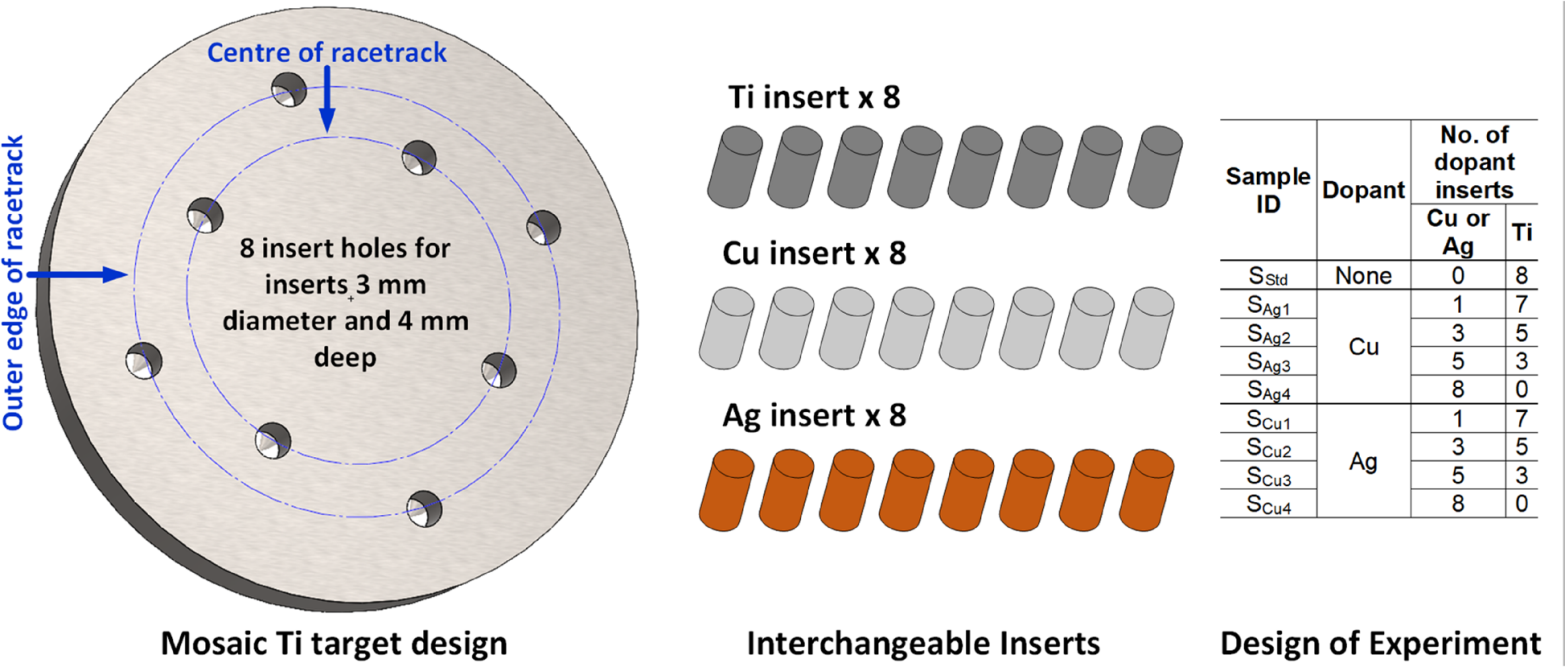
Schematic of custom-made mosaic Ti sputtering target with 8 holes to accommodate Ag and Cu doping inserts and a table showing design of experiment to explore the effect of dopant concentration in Ti_3_Au thin films by varying the number of Ag and Cu inserts in the custom-made mosaic Ti target

While antimicrobial coatings can be synthesized by various methods such as chemical, physical or biological, (see comprehensive study in our previous work [37]), this study employed sputtering as its choice of synthesis technique, due to its precise control over chemical composition and ability to form uniform dense thin films over a large surface area. The magnetron sputtering technique was used to deposit all of the Ti_3_AuX (x = Ag, Cu) thin films samples onto glass slides and Ti_6_Al_4_V medical grade substrates, using a S10A Nano-PVD sputtering system from Moorefield Nanotechnology Ltd, UK. Ti_6_Al_4_V strips (76 × 25 × 1 mm^3^) were polished using SiC paper (P240 to P4000) on a wheel polisher to a surface finish better than 40 nm, verified in either direction using an Alicona Infinity Focus surface measurement system. The polished Ti_6_Al_4_V strips were then cut into smaller substrates (19 × 25 mm^2^) and cleaned, together with standard microscopic glass slides (76 × 25 × 1 mm^3^), using 5:1 solution of water to Decon 90 cleaning agent, followed by ultrasonic bath and rinse in isopropanol (IPA) and acetone to remove surface adherents. After a second ultrasonic bath, the substrates were blown dry using dry nitrogen and loaded into the deposition chamber at a distance of 100 mm from the plasma source and rotated at a constant speed of 5 rpm during the deposition process. The deposition system was fitted with two magnetrons, set in co-sputtering mode to achieve simultaneous deposition of two target materials to form intermetallic compounds. One magnetron was loaded with a 50.8 mm diameter by 6.35 mm thick mosaic Ti target and connected to a DC power source and the other was loaded with a standard Au target of the same dimensions and connected to an RF power source. The mosaic Ti target was custom built by Pi-KEM Ltd, UK, with 8 blind holes (3 mm diameter × 4 mm deep) on the sputtering surface, to accommodate the Ag and Cu doping elements, see Fig. 1. In order to achieve better control of Ag/Cu doping concentration, these 8 blind holes were drilled in two sets with 4 in the centre of the sputtering target racetrack for a higher doping rate and 4 on the outer edge of racetrack for a lower doping rate. Additionally, 8 cylindrical target inserts (3 mm diameter × 4 mm long) of Ti, Ag and Cu, were supplied Pi-KEM, which can be readily interchanged between deposition runs to achieve thin film samples with varying dopant element concentrations. The power levels on the Ti and Au targets were precisely controlled to achieve the required 3:1 stoichiometric ratio between Ti and Au in each deposition run. The substrate surface temperature was heated to 450˚C and the working pressure was adjusted to 0.3 Pa to achieve better crystallization of the β phase of Ti_3_Au, in line with the findings in our previous work [1]. To explore the effect of a wide range of dopant concentration, 4 runs were conducted for each of the dopant elements (Ag and Cu) with 1, 3, 5 and 8 dopant inserts fitted in the mosaic Ti target and any remaining holes fitted with Ti inserts, see Fig. 1. The first dopant inserted was fitted into one of the holes in the outer edge of sputtering racetrack and thereafter alternating between the centre and outer edge of the racetrack. The electrical conductivity between each of the inserts and the mosaic target was verified to ensure a uniform sputtering plasma could be maintained across the entire target surface during deposition.

### Thin film characterisation

The crystal structure of the Ti_3_Au thin films doped with Ag and Cu ternary elements were studied using a Rigaku Smart Lab 2 X-ray diffractometer, equipped with Cu K_α_ radiation, in parallel beam configuration. The reflection patterns were collected for 2θ values between 10 to 80° and peak indexing was performed using the built-in crystallographic data base and confirmed with files from the ICSD (Inorganic Crystal Structure Database). The surface and cross section features of these samples were imaged using a Tescan Systems MIRA 3 scanning electron microscope (SEM), operating at 5 kV. Atomic concentration of each element within the thin film samples was analysed using an X-Max 150 energy dispersive X-ray (EDX) spectroscopy detector from Oxford Instruments, incorporated within the SEM. Elemental variation across the thin film thickness was verified using secondary ion mass spectroscopy (SIMS) from a Hidden Analytical gas ion gun (argon) rastered over a 500 × 500 µm^2^ area and a quadruple detector. Lamellae for transmission electron microscopy (TEM) were milled using a FEI Helios Nano Lab 600 Dual Beam focused ion beam (FIB) system, operating a focused 30 keV Ga liquid metal ion source. TEM images were obtained using a JEOL 2100F TEM system operating at 200 keV with an Oxford Instruments micro-analysis system running at 200 keV and 0.7 nA in scanning transmission operation to perform cross section micro-analysis. The surface roughness of the films was measured from atomic force microscope (AFM) scans generated using a Nanoveeco Dimension 3100 AFM fitted with a 15 nm sharp silicon tip in continuous contact mode, rastering over a surface area of 3 × 3 μm^2^. The AFM scans were measured over 4 sample sites using Gwyddion processing software and the mean values of surface roughness were calculated. Mechanical properties were measured using a Bruker-Hysitron TI900-Triboindneter nanomechanical testing system fitted with a 3-sided Berkovich diamond tip. A set of 16 indents in a 4 × 4 pattern were made on each sample to a depth reaching close to 10% of the total film thickness. The resultant force displacement curves were then analysed according to the Oliver and Pharr indentation technique to extract hardness and elastic modulus values of the films from the unloading leg of the curve. The electrical conductivity of the thin film samples was calculated from the measurement of their sheet resistance using a T2001A3-EU four-point probe system (Ossila Limited, Sheffield, UK) and their thickness using a DektakXT stylus profilometer. Friction and wear behaviour were evaluated using a Nanovea T50 tribometer with AISI 440 stainless steel balls of 6 mm diameter sliding against the coating samples submerged in simulated body fluid (SBF) [38] at room temperature. A normal load of 1.52 N was applied to the stainless-steel ball when sliding in linear reciprocal motion mode at an amplitude of 5 mm and frequency of 1 Hz for 20 min, giving a total sliding distance of 12 m. Following the test, the wear scar morphologies were examined using an optical microscope of the Nanovea PB1000 Mechanical Tester, with coaxial white light illumination, and the wear volume was measured using a Nanovea JR25 profilometer. Electrochemical corrosion resistance measurements were conducted in a standard 3-electrode cell using a potentiostat/galvanostat workstation (Metrohm Vionic 3500001080) in SBF at 37°C, with the sample acting as the working electrode (2 cm^2^ exposed area), a platinum counter electrode and a silver/silver chloride reference electrode (Ag/AgCl). Prior to testing, the samples open circuit potential (OCP) was monitored continuously until stable conditions were attained. Potentiodynamic polarization measurements were then taken in the range -0.8 V to + 0.4 V at a scan rate of 1 mV/s and used to determine the anodic (*β*_*a*_) and cathodic (*β*_*c*_) Tafel slopes and corresponding values of corrosion current density (*i*_*corr*_), corrosion potential (*i*_*corr*_), polarization resistance (*R*_*p*_) and protective efficiency (*P*_*e*_) [39]. Electrochemical impedance spectroscopy (EIS) tests were performed in the frequency range 0.01 Hz to 100 kHz at an amplitude of 5 mV.

### Cell lines and culture reagents

L929 murine fibroblast cells were purchased from Deutsche Sammlung von Microorganismen und Zellkulturen (DSMZ – Braunschweig, Germany). Cells were cultured in Dulbeccos’s Modified Eagle Medium (DMEM) high glucose, supplemented with 10% fetal bovine serum (FBS), 2 mM L-glutamine, 100U/ml penicillin and 100 μg/ml streptomycin, and incubated under humidified conditions at 37 °C and 5% CO_2_. Murine fibroblast cells were grown as monolayer cultures and were sub-cultured for a maximum of 15–20 passages, before new vials were used. DMEM high glucose culture media and reagents [Phosphate Buffer Saline (PBS), foetal bovine serum (FBS), antibiotics and trypsin] were obtained from Biosera (Kansas City, MO, USA), while for cytotoxicity assays, Resazurin sodium salt was purchased from Fluorochem (Derbyshire, UK).

### Thin film extracts preparation

Extracts of thin film samples were prepared by following two distinct experimental protocols. Specifically, the first set of extracts was prepared through immersion of thin film test samples into 6-well culture plates containing 6 ml of DMEM media for 72 h in a humidified incubator at 37°C, 5% CO_2_. In addition, a slight agitation was applied to the 6-well plates containing the samples for 5 s at the beginning and the end of the preparation period, in order to promote ion leaching rates into the culture media, which were subsequently used for cytotoxicity experiments against cultured L929 mouse fibroblast cells. After the completion of the initial 72 h of leaching time, a second set of extracts from the same thin films was obtained by extending the immersion period by a further 96 h within the DMEM media. In addition to the thin film samples, extracts were also derived from polished Cu and Ti substrates to be used as positive and negative control samples, respectively, for the cytotoxicity experiments.

### Biocompatibility characterisation of thin films against L929 mouse fibroblast cells

#### Extract exposure method

The biocompatibility performance of the doped Ti_3_Au thin films was analysed by measuring their *in-vitro* cytotoxic effect through the Alamar blue assay and ion leaching potential according to ISO 10993 standards. Specifically, L929 cells were seeded at a density of 2000 cells/well into a 96-well culture plate and incubated overnight at 37°C and 5% CO_2_. The following day, DMEM culture media was aspirated and replaced with extracts obtained from thin films deposited on Ti_6_Al_4_V substrates, following 72 h and 168 h ion leaching periods. For each experimental condition, L929 cells were exposed to respective leached extracts for a total period of 72 h. Simultaneously, L929 cells were also exposed to both Cu substrate derived extracts and DMEM culture media containing 10% DMSO containing extracts, used as positive control conditions, while in negative control conditions, L929 cells were exposed to both complete DMEM culture media (Blank samples) as well as extract leached from a blank Ti substrate. Thereafter, following the completion of the 72 h exposure period of L929 cells with the above conditions, 10 μl of resazurin (1 mg/ml final concentration) was added to each well and cells were incubated for 4 h at 37°C. At the end of incubation period, absorbance measurements were performed at 570 and 590 nm (reference wavelength) using an absorbance plate reader (Labtech LT4500, UK) while cell viability levels were calculated and expressed as a percentage of untreated (Blank) cells.

Leached ion concentrations from Ti_3_Au thin films and blank Ti and Cu substrates into their respective extracts, were measured using a Perkin Elemer Optima 8000 inductively coupled plasma optical emission mass spectrometer (ICP-OEMS). A portion of the sample extracts prepared after 72 h and 168 h of leaching period, were passed through the mass spectrometer and the intensity of ion concentrations were compared against a set of pre calibrated standards containing 0.625, 2.5, 5.0 and 10 ppm of dissolved Ti, Al, V, Cu and Au.

#### Direct thin film surface exposure method

For immunofluorescence experiments, nucleus staining reagent 4′,6-diamidino-2-phenylindole (DAPI) was purchased from Sigma-Aldrich Co. (Taufkirchen, Germany), fluorescent mounting medium was from DAKO (S3023), Phalloiding CF®440 antibody was from Biotium (Fremont, CA, USA), while microscope cover glasses were purchased from Paul Marienfeld GmbH & Co. KG (Lauda-Königshofen, Germany).

For immunofluorescence analysis, L929 fibroblast cells were seeded on glass coverslips inserted into 100 mm culture dishes and allowed to grow overnight. The following day, the different thin film test samples were placed into 100 mm plates and fibroblast cells were directly incubated for a total of 72 h. In parallel, mouse fibroblasts were also incubated with Cu substrates and DMEM culture media containing 10% DMSO, used as positive control conditions, while for negative control conditions, both blank Ti substrates and complete DMEM culture media (Blank samples) were used. At the end of treatments, cells were initially washed three times with PBS and were then fixed on ice with 3.75% formaldehyde in PBS for 15 min. Following three wash cycles with PBS, cells were permeabilized by adding PBS, pH 7.4 containing 0.5% Triton-X for 10 min. Cells were subsequently washed three times for 5 min with PBS and incubated with fluorescent Phalloidin antibody at room temperature for 20 min. After that cells were washed three times with PBS, while cell DNA was counterstained with DAPI (1 μg/mL) for 5 min, washed with PBS and finally coverslips were mounted with fluorescent mounting medium prior to observation. Cell images were obtained with a 40X lens and a fluorescence microscope (Zeiss Axionvision software, Carl Zeiss Microimaging, Oberkochen, Germany), was used for image acquisition. For images analysis, the Image J software was used.

For cytotoxicity analysis following direct incubations of L929 cells on the samples detailed above, the thin films and glass cover slips were removed from the 100 mm culture plates and cells from each condition were collected via trypsinization. Subsequently cells were centrifuged, resuspended in DMEM culture media, counted and 5000 cells/well were seeded into a 96-well culture plate and incubated at 37 °C and 5% CO_2_ overnight, for Alamar blue cytotoxicity assay. Moreover, in order to determine the effect of direct thin film exposures, a cell viability measurement was performed on the same day using the Trypan blue exclusion assay. Specifically, equal (10 μl) parts of L929 cells from each of the above conditions and 0.4% Trypan blue stain were mixed, live and dead cells were counted and the percentage (%) of viable cells was calculated using the following formula: viable cells (%) = total number of viable cells per ml/total number of cells per ml × 100. Finally, the following day, the Alamar blue cytotoxicity assay was performed, as a comparative study to that of the extract exposure method detailed earlier, in terms of cell viability levels, expressed as a percentage of untreated (Blank) cells.

### Antimicrobial test

*E. coli* TOP10 cells expressing the recombinant *ilux* gene cassette [40] were cultured aerobically at 37°C in LB broth containing a final concentration of 100 μg/ml Ampicillin (Amp). After 24 h of growth, the cells were harvested by centrifugation at a g-force of 3200 g for 15 min. The cells were washed by resuspension in 10 ml fresh LB/Amp. The centrifugation was repeated, and the resulting pellet of cells was resuspended in 5 ml of fresh LB/Amp. These cells in aliquots of 5 ml with a cell density of 6 × 10^9^ cfu/ml, were then used for analysis of the thin film surface’s antimicrobial properties.

The thin film samples were situated and fixed by adhesive tape on their rear side to a glass plate to retain their positioning during the analysis. Aliquots (5 ml) of the cell suspension were added to the centre of each sample surface and placed inside a GeneSys (Syngene) GBox with no illumination for image capture. GeneTools (Syngene) software was programmed for an exposure time of 3 min i.e., to take images of the individual 5 μl droplets after 1, 4, 7, 10, 13, 16, and 19 min. All experiments were performed in triplicate. These images were exported as TIFF images and analysed by the visual identification of bioluminescent dots. All dots were selected individually using the software selection tool along with an appropriate background sample to act as a blank reading. Following this manual identification, the light intensity results were exported to Microsoft Excel for further analysis.

Each individual spot had the background light intensity removed and the triplicates used to calculate an average light intensity for each surface sample and time point. The logarithmic values of the averaged bioluminescence readings were then plotted against time to produce a linear trendline relationship between bioluminescence intensity and time. This allowed us to produce an equation of the straight line and an R^2^ value to measure the fit.

## Results

### Structural and chemical characterisation

The elemental composition of Ti_3_Au thin films doped with Cu/Ag measured using EDX technique, along with their cross-section thickness are presented in Table 1. The table compares the stoichiometric Ti:Au ratio in Cu/Ag doped Ti_3_Au thin films against the Ti:Au ratio of 2.7:1 (73.3:26.7 at%) registered for the standard undoped Ti_3_Au thin film. It can be seen that all of the Ti_3_Au thin films doped with Cu/Ag never deviate by more than 0.3 from the required Ti:Au ratio of 3. The level of Cu doped in Ti_3_Au thin films varies from 0.5 to 7.1 at% whereas the Ag level ranges from 0.2 to 4.2 at%. The higher sputter rates of Ag/Cu doping elements is evident from the increase in the film thickness observed with increasing number of target inserts. Cu doped Ti_3_Au films register an increase from 900 nm with 1 insert to 1130 nm when the number of inserts is increased to 8. Similarly, the Ti_3_Au films doped with Ag increase from 1220 to 1560 nm with increase in number of dopant inserts from 1 to 8. The relative increased thickness of Ag doped samples compared to Cu doped ones is due to agglomeration of Ag particles on the film surface, as shown later in the SEM images in Surface and cross section characterisations section.

**Table 1:** EDX elemental composition and thickness for Ti_3_Au films doped with Cu and Ag elements

Sample ID	EDX analysis (at%)				Film thickness (nm)
	**Ti**	**Au**	**Cu or Ag**	**Ti:Au ratio**	
**S**_**std**_	73.3	26.7	-	2.7	915
**S**_**Cu1**_	75.8	23.7	0.5	3.2	900
**S**_**Cu2**_	72.2	26.6	1.2	2.7	1000
**S**_**Cu3**_	71.8	25.0	3.2	2.9	1120
**S**_**Cu4**_	71.2	21.7	7.1	3.3	1130
**S**_**Ag1**_	75.7	24.1	0.2	3.1	1220
**S**_**Ag2**_	74.8	23.9	1.3	3.1	1070
**S**_**Ag3**_	70.9	26.3	2.8	2.7	1250
**S**_**Ag4**_	70.7	25.0	4.2	2.8	1560

**Fig. 2:**
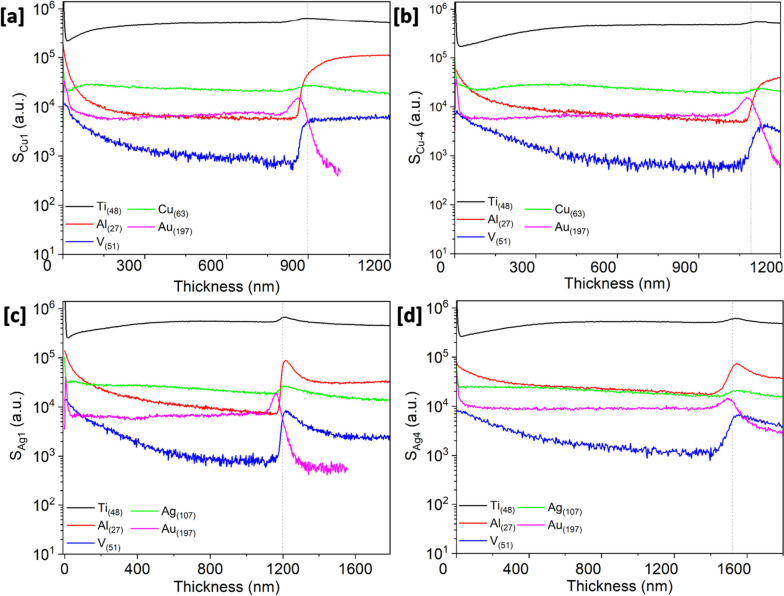
SIMS depth profiles showing variation in Ti, Al, V, Cu, Ag and Au ions through sample **a** S_Cu1_ with 0.5 at% Cu, **b** S_Cu4_ with 7.1 at% of Cu, **c** S_Ag1_ with 0.2 at% Ag, and **d** S_Ag4_ with 4.2 at% of Ag

SIMS analysis was performed on the Ti_3_Au films with the lowest and highest concentration of Cu (S_Cu1_ and S_Cu4_) and Ag (S_Ag1_ and S_Ag4_) dopants and depth profiles for each element are shown in Fig. 2. The SIMS results are used to convey the relative variation of each individual element with the depth of thin film but unlike EDX measurement, it is not a representation of relative concentration between elements and therefore cannot be used to compare stoichiometry of the thin film. In all four films, Ti [black line] registers a steady profile throughout the film thickness, with a small increment at the film-substrate interface (vertical dotted line). Other main components of the Ti_6_Al_4_V substrate, Al [red line] and V [blue line] peak sharply around the film-substrate interface. Au [purple line] intensity follows the opposite trend being consistent throughout the thin film and dropping sharply at the film-substrate interface. Cu doped films (Fig. 2a and b) exhibit a steady presence of Cu [green line] throughout the thin film depth and similarly the films doped with Ag (Fig. 2c and d), show a steady presence of Ag [green line] throughout the film thickness. There is no discernible drop in Cu or Ag signals at the film-substrate interface due to their relatively low concentrations in the films. There is a 10% increment in the intensity of the Cu signal when moving from sample S_Cu1_ (Fig. 2a) to sample S_Cu4_ (Fig. 2b). Similarly, the intensity of the Ag signal increases by 7% between sample S_Ag1_ (Fig. 2c) to S_Ag4_ (Fig. 2d), but these changes are not clearly visible on the logarithmic scale. The increase in film thickness seen in profilometer measurements and cross section imaging is also supported in the SIMS profiles where, for example, sample S_Cu4_ having a thickness of 1100 nm, took longer to sputter etch compared to S_Cu1_ with a thickness of 900 nm.

**Fig. 3:**
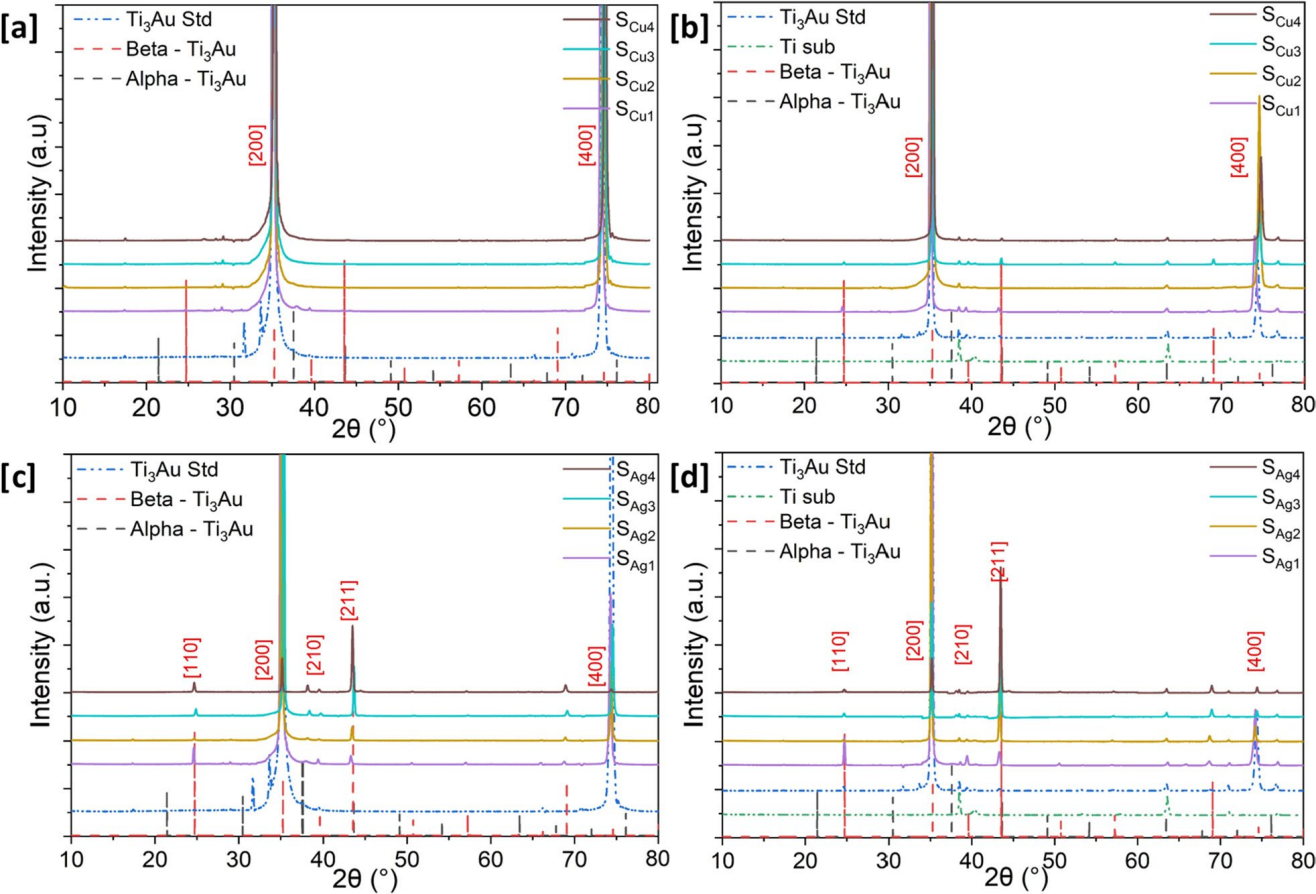
X-ray diffraction patterns for Ti_3_Au thin films deposited with varying amounts of Cu doping on **a** glass substrate **b** Ti_6_Al_4_V and for Ti_3_Au thin films deposited with varying amounts of Ag doping on **c** glass substrate **d** Ti_6_Al_4_V substrate

Diffraction patterns of Cu/Ag doped Ti_3_Au thin films deposited on glass and Ti_6_Al_4_V substrates are presented in Fig. 3a to d. Stoichiometric addition of Au in the Ti leads to formation of Ti_3_Au intermetallic at a ratio of 3:1. This composition when deposited at elevated temperature of 450˚C leads to emergence of the β phase of this intermetallic. The peak position of the standard Ti_3_Au thin film [dashed blue line] shows an excellent match to the single phase β-Ti_3_Au intermetallic with preferential orientation along [200] and [400] directions (ICSD 58605 – dotted red line). On glass substrate, the Ti_3_Au thin film samples doped with Cu present main peak positions at 35.27˚ and 74.59˚ which shows presence of a well-defined β-Ti_3_Au intermetallic phase with preferential orientation along the {100} family of planes, in very good agreement with both standard Ti_3_Au film patterns as well as standard ICSD peak positions. The diffraction patterns for the Cu doped Ti_3_Au thin film samples deposited on Ti_6_Al_4_V substrates also suggest the presence of preferentially oriented β-Ti_3_Au. However, at higher Cu concentration (S_Cu4_), the intensity of the (400) plane is reduced and the peak position is shifted to a higher angle, indicating that Cu ions are alloying into the Ti_3_Au matrix causing lattice distortions and in plane stress [41]. Apart from some very minor peaks for Cu based intermetallic like Ti_3_Cu_4_ (ICSD 629390) and TiCu_4_ (ICSD 629385) observed at 17.4˚ and 29.2˚ respectively, no other Cu peaks were observed. Cu (1.28 Å) has a smaller ionic radius when compared to Ti (1.47 Å) [42, 43] and therefore causes the changes in the crystal structure leading to a decrease in diffraction peak intensity and a shift to a higher angle [41].

On the other hand, addition of Ag leads to visible changes in the Ti_3_Au thin film diffraction pattern and therefore more significant changes in the structural arrangement. Preferential orientation along [200] and [400] directions observed in the standard Ti_3_Au thin film sample is disturbed with addition of Ag and new peaks positioned at 24.7˚, 39.6˚, 43.5˚ and 68.9˚, representative of (110), (210), (211) and (312) planes of the β-Ti_3_Au intermetallic phase, emerge. This is accompanied by a very steep reduction in intensity of the [200] and [400] peaks. For Ag rich thin films (SA_g3_ and S_Ag4_), the [211] peak dominates, and one minor peak emerges around 37.9˚, aligning with the [111] orientation of the α-Ti_3_Au intermetallic (ICSD 58604). Thin film samples deposited on Ti_6_Al_4_V substrates are similar to their glass counterparts and present a shift from the (200) to (211) plane with increasing Ag content in the Ti_3_Au films. However, no independent peaks for Ag or Ti-Ag intermetallics are present. The eutectoid point of the Ti-Ag and Ti-Cu intermetallics is expected above 15.6 and 7 at%, respectively, and therefore it is no surprise that no independent phase of Ti-Ag alloy is visible in the diffraction patterns and with the Cu concentration barely exceeding the required 7 at% limit, the minor phase of Ti-Cu alloy formed only registers a weak intensity [44–46]. The crystallite size of each thin film sample, calculated from the dominant diffraction peak using Scherrer’s equation [47] is presented in Table 2. With addition of Ag to the Ti_3_Au matrix, the crystallite size initially increases to 54.7 nm but then begins to reduce to 48.3 nm as the (211) plane becomes dominant. Whereas with increasing Cu concentration no such changes occur in crystal orientation in the Ti_3_Au matrix, and the average crystallite size steadily increases from 50.3 to 54.1 nm.

**Table 2:** Crystallite sizes for leading phases of Ti_3_Au thin films doped with Ag and Cu, calculated from their respective dominant diffraction peak using Scherrer’s equation

Sample ID	Crystallite size (nm)			
	**On glass sub**	**Std dev**	**On Ti sub**	**Std dev**
**S**_**std**_	49.8	0.1	47.2	0.1
**S**_**Cu1**_	50.3	0.1	46.2	0.0
**S**_**Cu2**_	51.8	0.1	46.1	0.1
**S**_**Cu3**_	52.3	0.1	49.8	0.1
**S**_**Cu4**_	54.1	0.1	48.4	0.1
**S**_**Ag1**_	49	0.1	43.9	0.1
**S**_**Ag2**_	53.2	0.1	55.5	0.4
**S**_**Ag3**_	54.7	0.1	53.3	0.1
**S**_**Ag4**_	48.3	0.2	36.5	0.2

### Surface and cross section characterisations

**Fig. 4:**
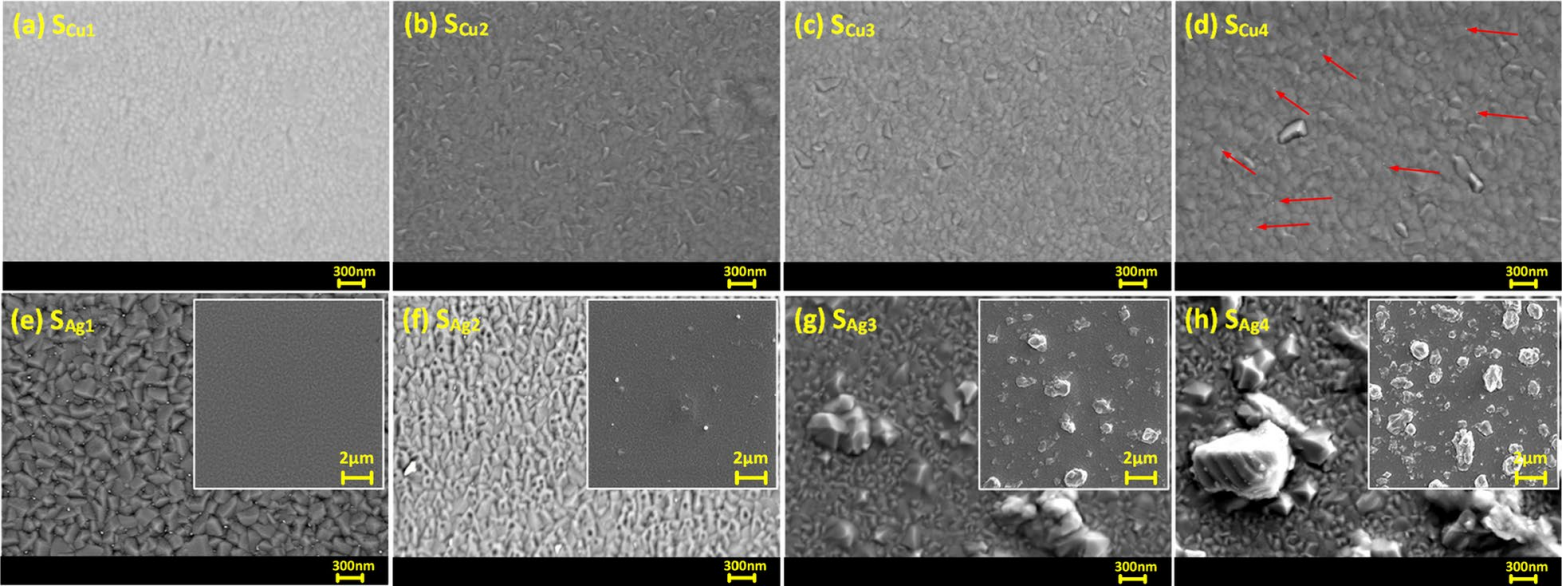
SEM surface images of Ti_3_Au thin films doped with varying Cu/Ag concentrations; **a** Sample S_Cu1_, **b** Sample S_Cu2_, **c** Sample S_Cu3_, **d** Sample S_Cu4_, **e** Sample S_Ag1_, **f** Sample S_Ag2_, **g** Sample S_Ag3_, and **h** Sample S_Ag4_. Inset images show increasing Ag agglomeration on the surface with increasing Ag concentration in film

SEM images of surface topology of Cu doped Ti_3_Au thin films are presented in Fig. 4a to d (surface SEM image of standard Ti_3_Au thin film provided in Supplementary data 1 for comparison). Magnetron sputtered pure Ti thin films are known to develop well oriented sharp pyramidal shaped grains whereas β-Ti_3_Au thin films develop extremely dense domes with angular edges [1, 4, 48]. The SEM images show that the smallest addition of Cu (S_Cu1_) causes development of light shaded spherical grains that are very well dispersed in the dark shaded Ti_3_Au matrix and such circular grains have previously been observed with addition of Cu in a Ti matrix [49]. With further increment of Cu in the matrix, this circular grain shape gives way to the development of elongated angular phases (S_Cu2_) which continue to grow with further increase in Cu doping (S_Cu3_ and S_Cu4_). Very faint agglomeration or out transport of Cu particles is visible in the sample with the highest Cu concentration of 7.1 at% (Fig. 4d – zoomed image available in Supplementary data 1) and supports the generation of Ti-Cu intermetallic above eutectoid point of 7at% inTi-Cu phase diagrams and is also supported by the XRD results seen earlier.

The surface topology of Ag doped Ti_3_Au thin films are presented in Fig. 4e to h, with low magnification inset images showing increasing Ag precipitation from the Ti_3_Au matrix. The Ti_3_Au thin film with the lowest Ag concentration (S_Ag1_) retains the more angular grain features of the pure Ti_3_Au thin film [4] but with some faint white spots visible (mean size = 43 nm, std dev = 10) on the edges, suggesting initial precipitation of Ag particles. But with further addition of Ag in the matrix (S_Ag2_), the angular grains transform into a flatter and cracked surface profile with increasing Ag agglomerations (mean size = 138 nm, std dev = 32) visible in between the features. This Ag agglomeration intensifies as more Ag is doped into the Ti_3_Au matrix, forming large Ag particles on the surface of sample S_Ag3_ (mean size = 700 nm, std dev = 150) and S_Ag4_ (mean size = 1018 nm, std dev = 80). Previous studies on Ag doped Ti-based alloys have demonstrated Ag precipitation with particle sizes reaching 200 nm for Ag concentrations as low as 9 at% in the film [50].

**Fig. 5:**
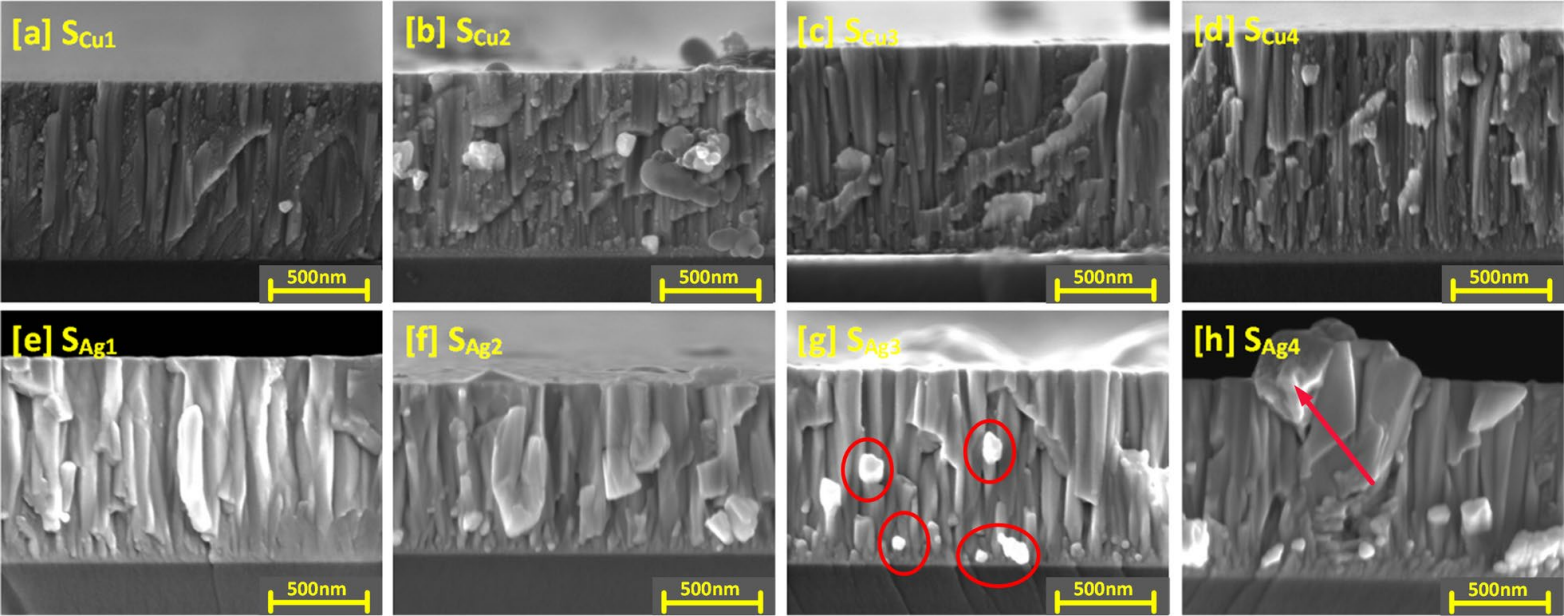
Cross sectional SEM images of Ti_3_Au thin films doped with varying Cu/Ag concentrations; **a** Sample S_Cu1_, **b** Sample S_Cu2_, **c** Sample S_Cu3_, **d** Sample S_Cu4_, **e** Sample S_Ag1_, **f** Sample S_Ag2_, **g** Sample S_Ag3_, and **h** Sample S_Ag4_. Figs **g**-**h** are highlighted to show increasing Ag agglomeration with increasing Ag concentration in film

The structure of the Cu doped Ti_3_Au thin films is further explored with the cross-section SEM images shown in Fig. 5a to d (cross section SEM image of standard Ti_3_Au thin film provided in Supplementary data 2 for comparison). For sample S_Cu1_, granular features can be observed, dispersed around the edges of the vertical columns, corresponding to the circular grains seen on the surface image of this sample. Formation of the Ti_3_Au phase is promoted by surface and intra-grain diffusion resulting from the high energy of incoming species when sputtered at very low deposition pressure and high substrate temperature [51]. This causes the incoming dopant species like Cu to be dispersed around the grain boundaries of the main phase leading to formation of such granular features at column boundaries. With further increases in Cu doping (S_Cu2_ to S_Cu4_), the spread of these granular features reduces, and the film structure becomes smoother, suggesting better diffusion of Cu from grain boundaries into the Ti_3_Au grains, resulting in formation of Ti-Cu intermetallic species. This observation is supported by presence of Ti-Cu peaks seen from XRD spectrum for this sample (Fig. 3) and from the granule development observed in the SEM surface images (Fig. 4).

Cross section SEM images of Ag doped Ti_3_Au films are presented in Fig. 5e to h and shed more light on the Ag precipitation observed in the surface profiles. The cross-section image of sample S_Ag1_ presents a well organised, tapered columnar structure with no observable Ag precipitation. With further increase of Ag concentration in sample S_Ag2_, angular Ag grains begin to grow out of the main Ti_3_Au columnar structure and can be clearly seen as bright Ag precipitates in sample S_Ag3_ between columns and on the surface in background. These precipitates continue to grow with further addition of Ag, leading to distortion of the Ti_3_Au columnar structure in sample S_Ag4_. Previous studies have shown that such Ag precipitation negatively affects thin film density, leading to variation in key performance properties like mechanical hardness and corrosion resistance [52].

**Fig. 6:**
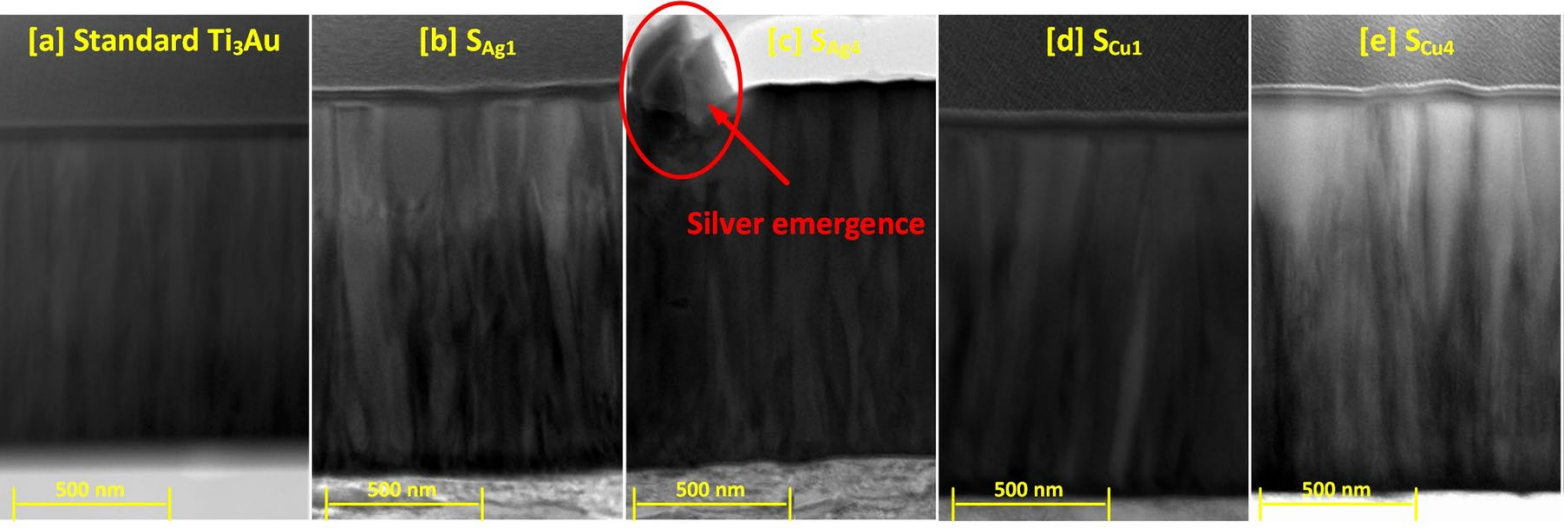
TEM images of **a** standard Ti_3_Au thin film in comparison to **b** sample S_Ag1_, **c** Sample S_Ag4_, **d** Sample S_Cu1_, and **e** Sample S_Cu4_

To further understand the cross-sectional microstructure of the thin films, TEM images were developed for the samples with lowest and highest concentration of Cu and Ag dopant elements, see Fig. 6a to e. For sample S_Ag1,_ presence of a small dopant concentration of Ag particles causes a wrinkled effect around the column boundaries disturbing the straight, dense, and well oriented columnar structure of the standard Ti_3_Au thin films. However, at higher Ag dopant concentration like in sample S_Ag4_, the agglomeration of silver becomes strong enough to precipitate out in between the column boundaries. On the other hand, the addition of Cu in low dopant concentrations like in S_Cu1_, causes no drastic change in the column structure and at the highest dopant concentration, Cu causes widening of Ti_3_Au thin film columns due to increased interdiffusion of Cu into the Ti_3_Au matrix, resulting in formation of Ti-Cu intermetallics or higher shift in angular position of the diffraction peaks (Fig. 3).

**Fig. 7:**
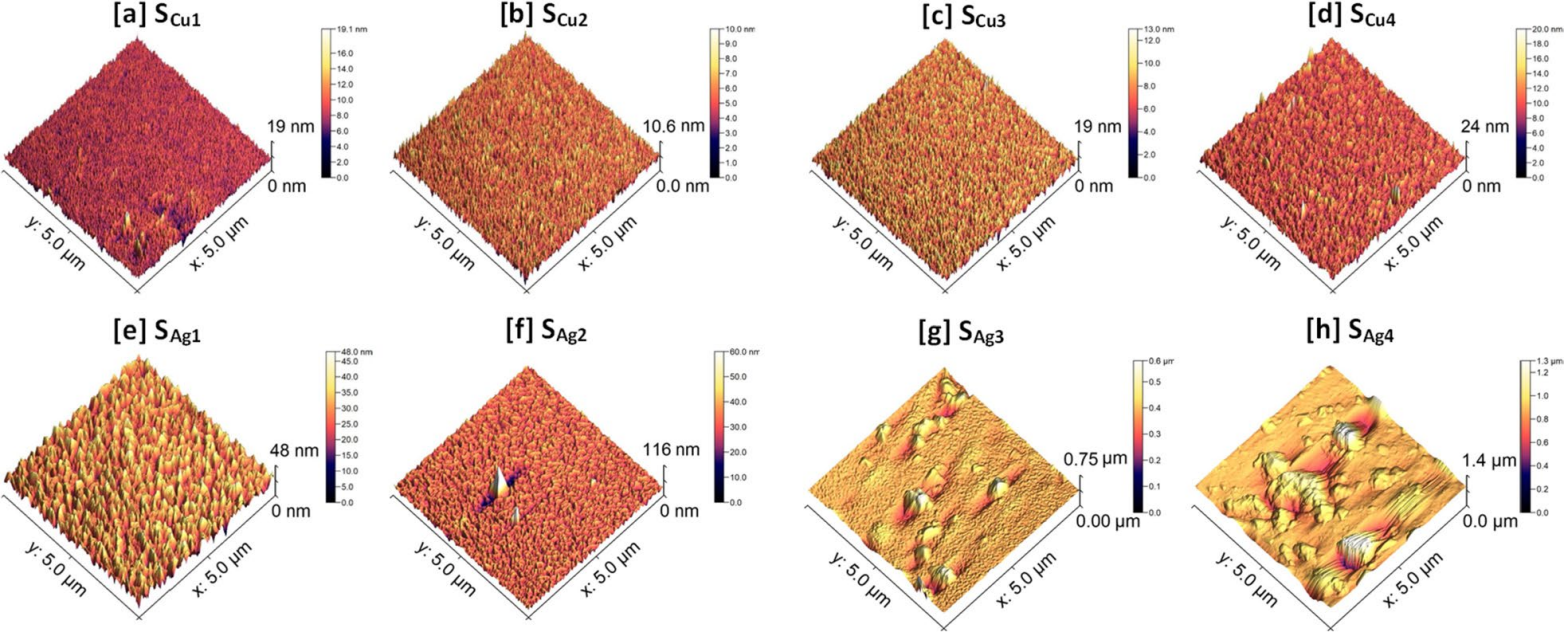
AFM surface profiles of Ti_3_Au films doped with **a** to **d** varying concentrations of Cu and **e** to **h** varying concentrations of Ag

The surface profiles of the Cu doped Ti_3_Au thin films were scanned using an AFM to investigate their feature sizes and mean surface roughness, see Fig. 7a to d (AFM profile for standard Ti_3_Au thin film provided in Supplementary data 2 for comparison). Sample S_Cu1_ registered a surface profile with feature sizes reaching up to 19 nm but this reduced to 10.6 nm with increasing Cu concentration in sample S_Cu2_. These results are in good agreement with the circular grainy features visible on the surface of S_Cu1_ (Fig. 4a) compared to the smoother cracked surface observed for S_Cu2_ (Fig. 4b). Surface smoothening due to increased Cu concentration is also reflected in the measured surface roughness values, where S_Cu1_ registers a value of 0.99 ± 0.1 nm and S_Cu2_ has a reduced roughness of only 0.70 ± 0.01 nm. However, with further increase in Cu concertation, the maximum feature size grows again up to 19 nm for S_Cu3_ and 24 nm for S_Cu4_, with corresponding increases in surface roughness values to 1.09 ± 0.03 nm and 1.13 ± 0.05 nm, respectively. The elongation of grains for sample S_Cu4_, seen previously in the SEM image (Fig. 4d) is also visible in the AFM scan. These surface AFM scans are very uniform with low surface roughness values and support the SEM observation that there is no precipitation of Cu particles or agglomerations on the film surfaces.

AFM scans of Ti_3_Au thin films doped with Ag are presented in Fig. 7e to h. When compared to the Cu doped films, addition of Ag leads to the formation of larger feature sizes on the film surface. Sample S_Ag1_, with the lowest Ag concentration, registers feature sizes of 48 nm with a mean surface roughness of 4.4 ± 0.1 nm. However, with further addition of Ag in sample S_Ag2_, the feature size increases abruptly to 116 nm, and can be correlated to the presence of a few large bright white coloured particles on the scanned surface, suggesting precipitation of Ag from the Ti_3_Au matrix is taking place. The measured surface roughness of sample S_Ag2_ is 3.5 ± 0.35 nm, which is similar to that of S_Ag1_. With further increase in Ag doping concentration, the feature sizes increase significantly to a maximum value of 0.75 μm due to the presence of large Ag particles and agglomerates on the film surface. And finally for sample S_Ag4_, the maximum feature height extends up to 1.4 μm, while also growing in the radial direction and merging with neighbouring Ag granules to form larger particles. Samples S_Ag3_ and S_Ag4_ register mean surface roughness values of 16.1 ± 2.5 nm and 54.8 ± 14.3 nm, respectively. Thus, from the results in this section it can be concluded that agglomeration of Ag particles plays a significant role in moderating the crystal orientation, grain shape, cross sectional structure, density, and surface roughness of the Ti_3_Au thin films.

### Mechanical characterisation

**Fig. 8:**
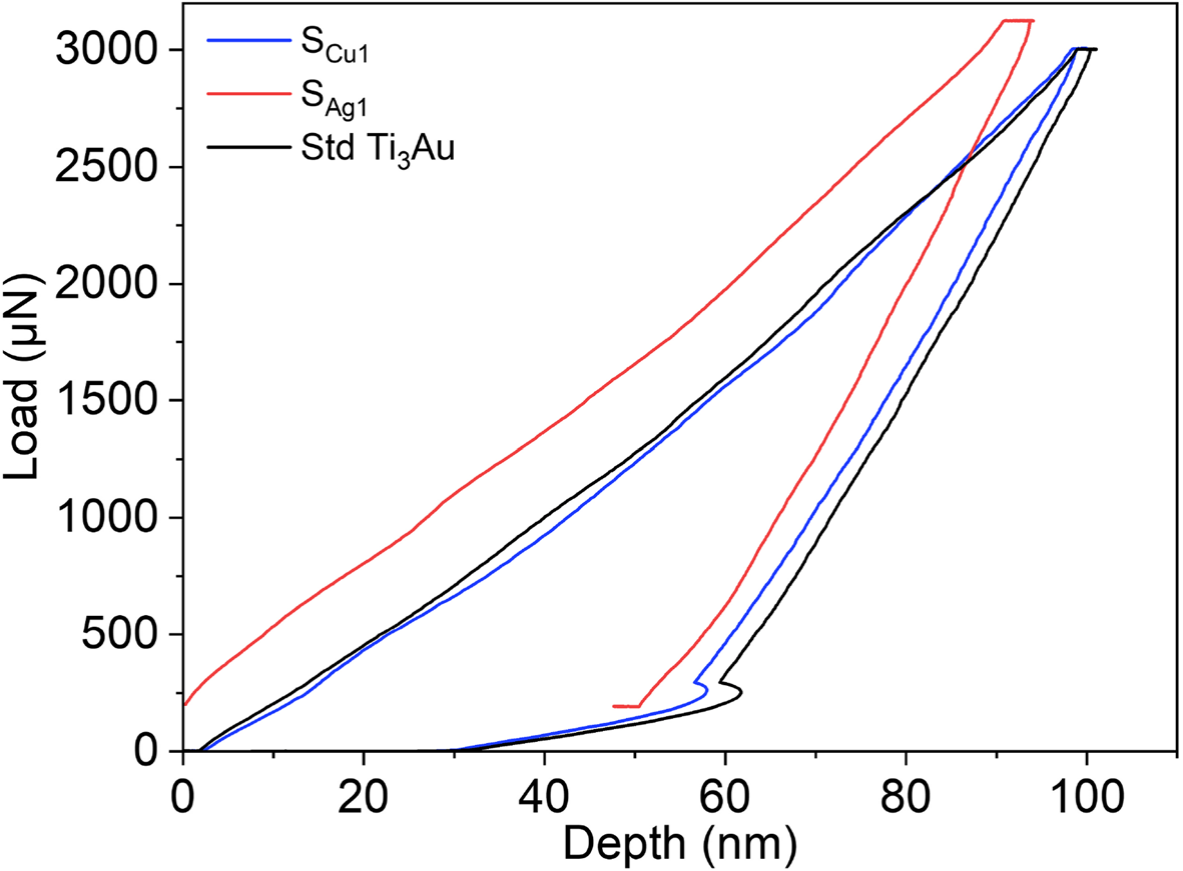
Load displacement curves for Ti_3_Au films doped with Ag (SAg1) and Cu (SCu1), compared against that of the standard Ti_3_Au film

**Fig. 9:**
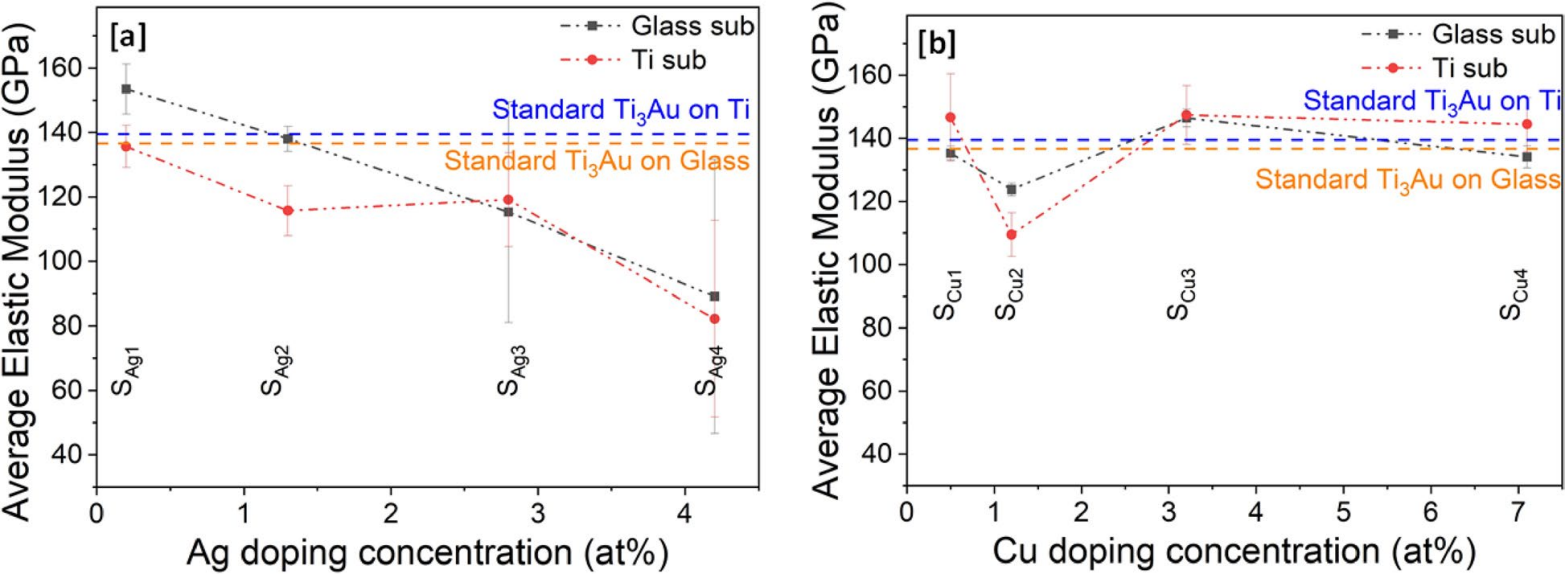
Variation in reduced elastic modulus (Er) of Ti_3_Au thin films deposited on Ti and glass substrates with varying **a** Ag and **b** Cu doping concentration

The load displacement (P–H) curve derived from a nanoindentation test gives information about the interaction of the diamond indenter tip with the thin film material beneath it and Fig. 8 compares the P–H curves generated by indenting the Ti_3_Au samples doped with the lowest concentration of Ag and Cu, against that of a standard Ti_3_Au thin film. The curves presented are all generated from indentations made at a constant peak load of 3000 μN and with load-dwell-unload time durations of 10 s each. Occurrence of displacement excursion more commonly known as “staircase phenomenon”, can be ruled out because of the absence of any sudden shift along the x-axis for any of the segments of the P–H curves [53, 54]. Displacement excursion take place when the indenter tip encounters any surface contaminants, phase difference or the presence of oxides with differing mechanical hardness, leading to unusual variation in displacement with increasing load [55]. The extensive substrate cleaning process used ensures removal of surface contaminants and in-situ processing avoids oxide formation at high temperature. XRD patterns (Fig. 3) only show the presence of single phase β-Ti_3_Au when the films are doped with Ag and very minute quantities of α-Ti_3_Au at higher Cu concentration of 4.2 at%. The presence of softer Ag agglomerations within the Ti_3_Au matrix, as seen in SEM images (Figs. 4 and 5), leads to a larger distribution in the mechanical hardness measurements (as explained below) but the P–H curve remains smooth and event free all throughout the nanoindentation cycle (Fig. 9) [56]. For the standard Ti_3_Au thin film (black line), the indenter tip travels to a depth in excess of 100 nm at the peak indentation load of 3000 μN and then elastically recovers to a calculated contact depth of 70.7 nm when the load is removed. Analysing the unloading curve segment according to the Oliver and Pharr method [57], gives a reduced elastic modulus value of 138.6 GPa and a mechanical hardness of 12.3 GPa. While for sample S_Cu1_ (blue line) with a Cu dopant concentration of 0.5 at%, the indenter tip travels to a slightly reduced maximum depth of 96 nm at peak load and returns to a contact depth of 69 nm when the load is removed. This curve translates to a harder thin film with mechanical hardness of 12.7 GPa and elastic modulus of 143 GPa. Sample S_Ag1_ (red line) doped with 0.2 at% of Ag offers the most resistance to tip penetration, only reaching a maximum depth of 91 nm and then elastically recovering to a contact depth of 66 nm. The elastic modulus and mechanical hardness calculated for this sample are 157 GPa and 14 GPa, respectively. The area enclosed by the load-unload hysteresis curve accounts for the work done by indenter tip to leave a plastic deformation on the film surface, whereas the hysteresis of the unloading curve is caused by the inherent elasticity of the thin film under test [58, 59]. To further understand the effect of Ag and Cu doping on the mechanical properties of the Ti_3_Au films, a set of 16 indentations, in 4 × 4 pattern was performed on each sample and the averaged results are discussed below.

Results for reduced elastic modulus results generated from nanoindentations performed on Ti_3_Au thin films doped with Ag and Cu on glass and Ti substrates are presented in Fig. 9a and b. The elastic modulus for the Ti_3_Au sample doped with the lowest Ag concentration (S_Ag1_) on glass was 135 ± 6 GPa, which is very close to the value of 136.7 ± 3 GPa achieved for the standard Ti_3_Au thin film on glass. However, with further addition of Ag in the Ti_3_Au film, the value of elastic modulus follows a downward trend with values of 115 ± 7, 119 ± 14 and 82 ± 30 GPa for samples S_Ag2_, S_Ag3_ and S_Ag4_, respectively. The error bar associated with sample S_Ag3_ is almost double that of S_Ag2_, while for S_Ag4_, the corresponding variation in results is over four times that of the samples doped with lower Ag concentrations. This increase in error bar can be attributed to agglomeration of Ag at higher concentrations, forming a uniform distribution of large Ag particles across the sample surface. When the indenter tip contacts the Ti_3_Au film it records a higher elastic modulus but if the tip encounters a Ag particle on the surface the recorded elastic modulus is very low, thus leading to a wider variation in results for the 16 indentations in the 4 × 4 pattern. It has been proposed that the elastic moduli of an alloy changes in proportion to the number of solute atoms when the alloying element concentration is low [44–46]. The Young’s modulus of Ag is reported to be 73 GPa, which is much lower than that of Ti at 112 GPa [44, 60]. XRD patterns (Fig. 3) showed that the Ag doping concentration of 0 to 4.2 at% is lower than that required to form any Ti-Ag intermetallic compound and thus, Ag exists as a solid solute within the Ti_3_Au matrix, leading to a decrease in the elastic modulus of the Ti_3_Au-Ag compound with increasing Ag concentration in the thin film [44–46]. Ti_3_Au samples doped with Cu report elastic modulus values very close to the value observed for the standard Ti_3_Au thin films (Fig. 9b). The elastic modulus of Cu doped samples deposited on glass remain between 144 to 147 ± 8 GPa with a dip in the trend for sample S_Cu2_, reporting a modulus value of 109 ± 6 GPa. A similar result is seen for Cu doped samples deposited on Ti substrates with elastic modulus values between 134 to 146 ± 3 GPa and a dip in the curve to 123 ± 3 GPa for sample S_Cu2_. Cu is reported to have a Young’s modulus of 128 GPa, which is greater than that of Ti (112 GPa) and therefore a positive effect is expected with addition of Cu in the Ti_3_Au matrix [60]. Further, the precipitation of Ti-Cu intermetallic compounds at the lower Cu concentration of 7 at%, leads to stiffening of the Ti_3_Au thin film matrix with increasing Cu concentration [44, 46, 61]. The observed stabilization of elastic modulus values for Cu dopant concentrations above 2.8 at% can also be explained due to grain refinement and the constraining effect of neighbouring grains [62].

**Fig. 10:**
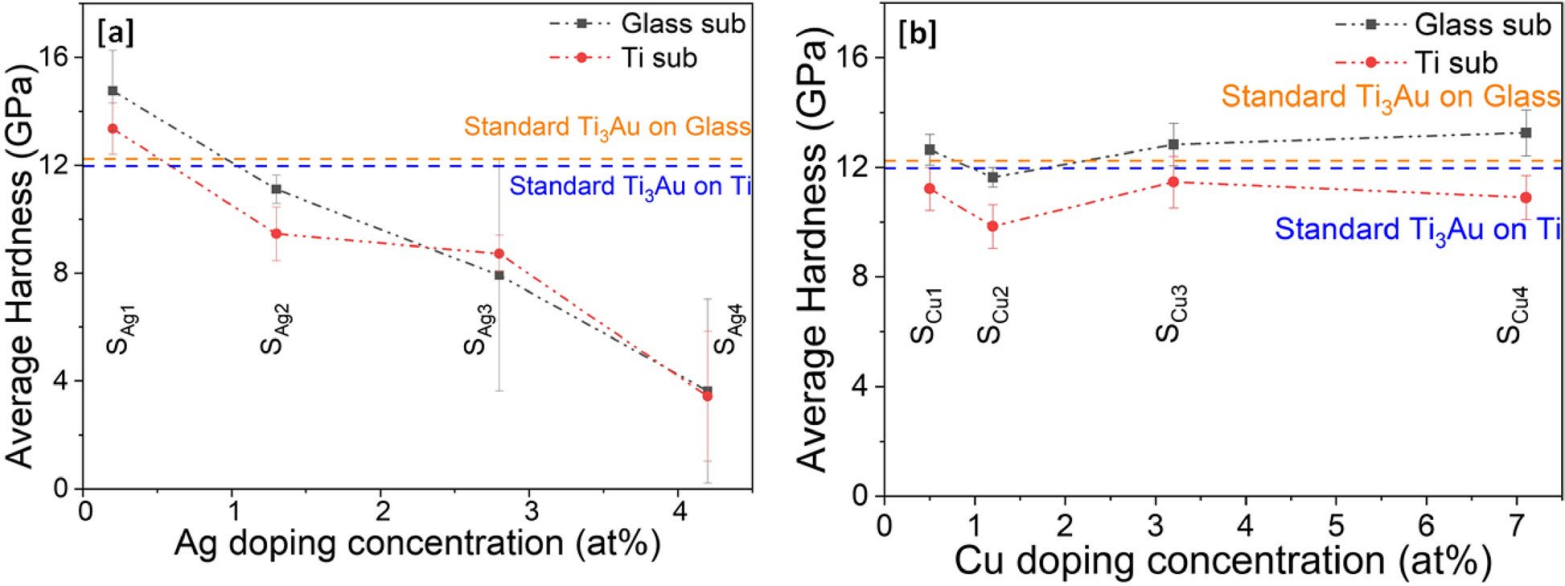
Variation in mechanical hardness (H) of Ti_3_Au thin films deposited on Ti and glass substrates with varying **a** Ag and **b** Cu doping concentration

Results of mechanical hardness extracted from nanoindentations performed on Ag/Cu doped Ti_3_Au thin films on glass and Ti substrates are presented in Fig. 10a and b. It can be observed that hardness follows a very similar trend to that seen for elastic modulus in Fig. 9. For samples doped with Ag, the hardness values steadily decrease with increasing Ag content in film. Sample S_Ag1_, doped with lowest Ag concertation of 0.2 at% reports excellent hardness values of 14.7 ± 1.4 and 13.3 ± 0.9 GPa on glass and Ti substrates, respectively, exceeding the values achieved for the standard Ti_3_Au thin films. With further addition of Ag in sample S_Ag2_, the hardness values dip below that of the standard Ti_3_Au to 11.1 ± 0.5 and 9.9 ± 0.9 GPa on glass and Ti substrates, respectively. However, with higher Ag addition, for samples S_Ag3_ and S_Ag4_, the hardness values decrease even further with a large variation in the spread of results. This increase in error bar size could be assigned to severe agglomeration of Ag from the Ti_3_Au matrix, causing the nanoindenter tip to indent on both soft Ag and hard Ti_3_Au matrix regions of the films. It is therefore safe to say that only the Ag dopant concentration of 0.2 at% (S_Ag1_) is useful to extend the mechanical hardness of the standard Ti_3_Au thin film coating. On the other hand, the Cu doped Ti_3_Ag thin films maintain a better hardness profile throughout the entire doping concentration range of 0.5–7.1 at%. Samples S_Cu1_, S_Cu3_ and S_Cu4_ deposited on glass substrates present similar hardness values of 12.6 ± 0.5, 12.8 ± 0.7 and 13.2 ± 0.8 GPa, respectively, which are all slightly higher than the standard Ti_3_Au thin film result. While sample S_Cu2_ presents a slightly lower mechanical hardness of 11.6 ± 0.3 GPa. The Cu doped samples deposited on Ti substrates present hardness values of between 9.8 to 11.4 ± 0.9 GPa, which are all slightly lower than the standard Ti_3_Au thin film result.

**Fig. 11:**
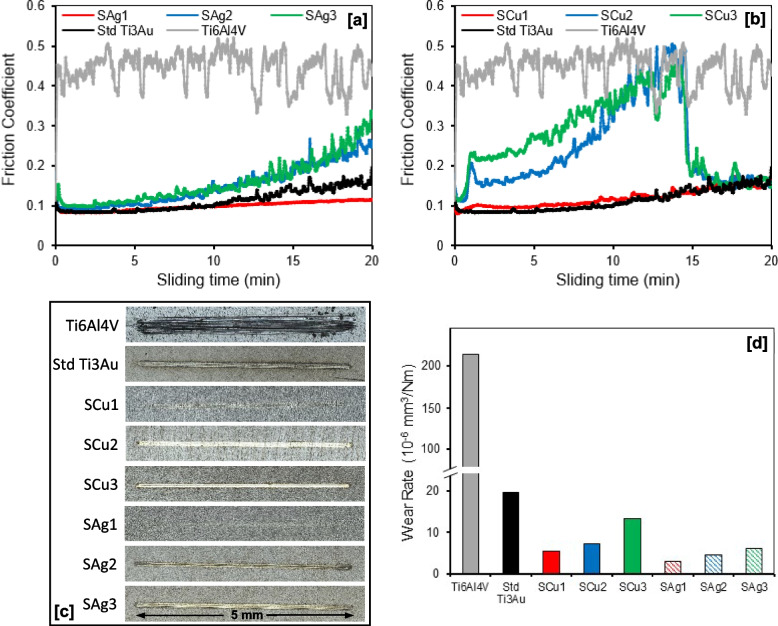
**a** and **b** Friction coefficient curves; **c** Wear track surface morphologies; and **d** Specific wear rates, for Ti_3_Au thin films deposited on Ti_6_Al_4_V substrates with varying Ag/Cu dopant concentration, when sliding against 6 mm diameter stainless steel balls at applied normal loads of 1.52 N in SBF solution at room temperature

Results for wear testing of the Ag and Cu doped Ti_3_Au thin films and Ti_6_Al_4_V alloy substrate are presented in Fig. 11, including their friction coefficients, wear track surface morphologies and specific wear rates when sliding against stainless steel balls at an applied normal load of 1.52 N in SBF solution. As shown in Fig. 11a, the friction coefficient of the Ti_6_Al_4_V substrate fluctuates around 0.4 to 0.5 throughout the 20 min of sliding time, whereas the standard Ti_3_Au thin film shows a significant reduction in value to ~ 0.1 for the first 10 min and gradually increases toward 0.2 in the second half of the test. Addition of a small quantity of Ag gives a further reduction in friction coefficient for sample S_Ag1_ to a consistent value of ~ 0.1 throughout the duration of the test, whereas further increments of Ag in samples S_Ag2_ and S_Ag3_ lead to increases in friction coefficient to final values around 0.25 and 0.3, respectively. A similar trend was observed for the Cu doped Ti_3_Au thin films (Fig. 11b), where addition of a small quantity of Cu in sample S_Cu1_ had minimal effect on the friction coefficient of the Ti_3_Au thin film, with a value < 0.2 throughout the full test duration. However, with further increments of Cu in samples S_Cu2_ and S_Cu3_, there was a noticeable change in the thin film behaviour, with a gradual increase in friction coefficients towards 0.5 in the first 15 min and then a sudden drop to < 0.2 for the final 5 min of the test. It is possible that in the first part of the tests the excess of doping material tends to create a third body that increases friction coefficient and wearing until the creation of a tribo-film that protects the surface by lowering the friction coefficient and the local roughness. This behaviour is commonly observed in thin film coatings doped with softer materials like Ag and Cu, which is normally caused by formation of a thin self-lubricating film on the coating surface. This reduces the adhesive trend between counterpart materials which in turn lowers the frictional coefficient and improves the wear performance [63–65].

In general, the reduction in friction coefficient of the Ag and Cu doped Ti_3_Au thin films compared to the Ti_6_Al_4_V substrate is associated with a decrease in real contact area and surface plastic removal due to their significantly higher surface hardness [66]. This result is reflected in the wear track surface images (Fig. 11c) and corresponding wear rate values (Fig. 11d), where the Ti_6_Al_4_V substrate has a much wider wear groove (~ 300 µm) than all the thin film samples and a significantly higher wear rate of 214.81 × 10^–6^ mm^3^/Nm. In comparison, the wear groove widths for the thin film samples are all < 100 µm and their corresponding wear rates are all < 20 × 10^–6^ mm^3^/Nm, which is less than 20 times lower than that of the Ti_6_Al_4_V substrate. As shown in Fig. 11d, addition of small quantities of Ag or Cu further reduces the wear rate of the standard Ti_3_Au thin film from 19.62 × 10^–6^ mm^3^/Nm to values of 3.03 × 10^–6^ and 5.50 × 10^–6^ mm^3^/Nm for samples S_Ag1_ and S_Cu1_, respectively. However, the wear rate increases with further additions of Ag and Cu in the Ti3Au thin film matrix, reaching values of 6.11 × 10^–6^ and 13.36 × 10^–6^ mm^3^/Nm for samples S_Ag3_ and S_Cu3_, respectively. This reduction in wear rate with addition of small quantities of soft elements like Ag or Cu can be related to trade-off between an increase in lubrication and a decrease in surface hardness [67]. Overall, results from this section suggest that addition of small quantities of antimicrobial elements like Ag and Cu can improve the mechanical performance of Ti_3_Au intermetallic thin films.

### Electrochemical corrosion tests

**Fig. 12:**
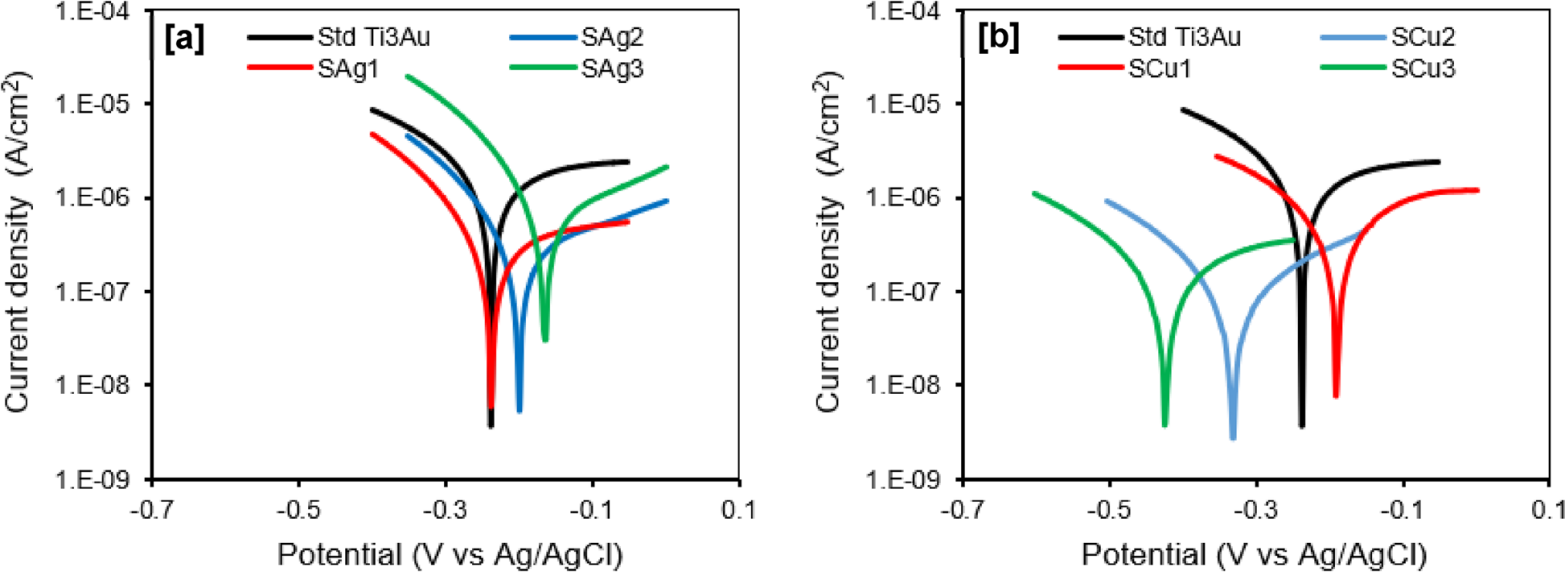
Potentiodynamic polarization curves for Ti_3_Au thin films deposited on Ti substrates with varying **a** Ag and **b** Cu dopant concentration, in SBF solution at 37 °C

An understanding of the surface coatings corrosion resistance is essential to evaluate its service life when applied to biomedical implants in the living tissue environment. Potentiodynamic polarization curves and corresponding corrosion parameters of the Ag and Cu doped Ti_3_Au thin films immersed in SBF at 37 °C are presented in Fig. 12 and Table 3, respectively. The corrosion resistance of the thin film samples can be examined on the basis of four evaluation criteria: (i) a more electropositive corrosion potential (*E*_*corr*_); (ii) a lower corrosion current density (*i*_*corr*_); (iii) a higher polarization resistance (*R*_*p*_); and (iv) a higher protection efficiency (*P*_*e*_) are indicative of an improved corrosion resistance [68]. As shown in Fig. 12a, the polarization curves of the three Ag doped Ti_3_Au thin films exhibit similar shapes and there is a shift towards more positive potential and a corresponding increase in current density with increasing Ag concentration in the films from S_Ag1_ to S_Ag3_. Conversely for the Cu doped Ti_3_Au thin films (Fig. 12b), the trend is opposite, with a shift towards more negative potential and a decrease in current density with increasing Cu concentration in the films from S_Cu1_ to S_Cu3_. As shown in Table 3, the values of current density (*i*_*corr*_) for Ag and Cu doped films are in the range ~ 0.1 to 0.7 µA/cm^2^, which is at least one order of magnitude lower than the value of ~ 1.7 µA/cm^2^ recoded for the standard Ti_3_Au thin film. For the Cu doped Ti_3_Au films, samples S_Cu2_ and S_Cu3_ exhibit the lowest values of *i*_*corr*_ of 0.107 and 0.175 µA/cm^2^, respectively. Whereas, for Ag doped Ti_3_Au films, samples S_Ag1_ and S_Ag2_ have the lowest values of 0.355 and 0.321 µA/cm^2^, respectively. This trend is also evident in the results for *R*_*p*_ and *P*_*e*_ shown in Table 3, where samples S_Ag1_, S_Ag2_, S_Cu2_ and S_Cu3_ all give higher values of polarization resistance and protection efficiencies of ~ 79 to 93% when compared to the standard Ti_3_Au thin film.

**Table 3:** Corrosion parameters obtained from potentiodynamic polarization curves of Ti_3_Au thin films deposited on Ti substrates with varying Ag/ Cu dopant concentration, in SBF solution at 37°C

Sample ID	*E*_*corr*_ (V_SCE_)	*I*_*corr*_ (µA/cm^2^)	*β*_*a*_ (V/decade)	*β*_*c*_ (V/decade)	*R*_*p*_ (kΩcm^2^)	*P*_*e*_ (%)
**Std Ti**_**3**_**Au**	-0.218	1.698	1.005	0.259	52.73	-
**S**_**Cu1**_	-0.194	0.694	0.755	0.270	124.59	59.13
**S**_**Cu2**_	-0.315	0.107	0.260	0.199	458.04	93.70
**S**_**Cu3**_	-0.425	0.175	0.564	0.219	391.92	89.69
**S**_**Ag1**_	-0.215	0.355	0.830	0.166	169.42	79.09
**S**_**Ag2**_	-0.166	0.321	0.364	0.164	153.14	81.10
**S**_**Ag3**_	-0.124	0.739	0.275	0.159	59.27	56.48

**Fig. 13:**
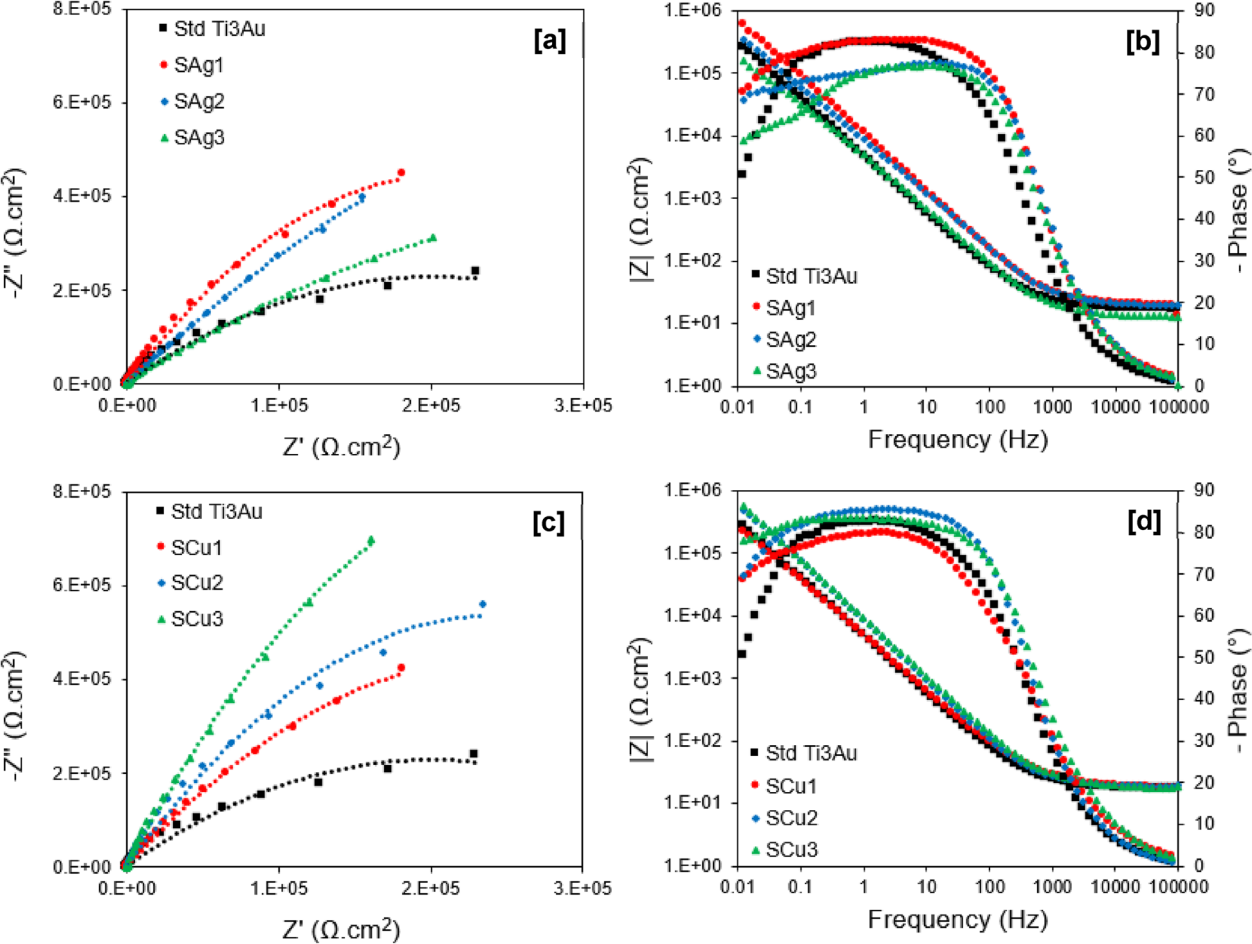
Nyquist plots and Bode plots for Ti_3_Au thin films deposited on Ti substrates with varying **a**-**b** Ag and **c**-**d** Cu dopant concentration, in SBF solution at 37 °C

These results are also supported by the EIS analysis shown in Fig. 13. For the Nyquist plots (Fig. 13a and c), a convenient and scientifically proven way to explain the electrochemical corrosion resistance of the thin film samples is to examine the diameters of the capacitive loops (semicircles), where a larger diameter signifies higher corrosion resistance [69] As can be seen, all samples exhibit a single capacitive loop, indicating a typical capacitive response of the passive thin films [70]. The diameters of the semicircles for the Ag and Cu doped films are larger than that for the standard Ti_3_Au thin film and increase with decreasing Ag (S_Ag3_ to S_Ag1_) and increasing Cu (S_Cu1_ to S_Cu3_) content in the film, denoting an improvement in corrosion stability, which is accordant with the potentiodynamic polarization results in Table 3. The Bode plots for the Ag and Cu doped films (Fig. 13b and d), also corroborate this finding. In the high frequency region, the impedance |Z| of all samples is low and independent of frequency value, and the phase angle is close to zero, indicating that impedance is dominated by the resistance of the SBF in this frequency range [70]. In the low and medium frequency region, the impedance |Z| of the samples increases linearly with decreasing frequency, with a slope of ~ -1, and the phase angle plateaus with a maximum value around -80°, which is a typical response of capacitive behaviour [71]. There is a notable decrease in the phase angle value and plateau width for samples S_Ag2_ and S_Ag3_ (Fig. 13b) in the midband frequency range, which could indicate a reduction in their electrochemical stability [66]. However, at low frequencies, the higher values of impedance |Z| for samples S_Ag1_, S_Ag2_ (Fig. 13b), and S_Cu2_, S_Cu3_ (Fig. 13d) suggest an improvement in electrochemical stability when compared to the standard Ti_3_Au thin film [72], and further support the results from the potentiodynamic polarization and Nyquist plots. Therefore, overall, the electrochemical corrosion tests have shown that small additions of Ag and Cu can improve the corrosion resistance of Ti_3_Au thin films in SBF solution at 37 °C and that the best performing films are S_Ag1_, S_Ag2_, S_Cu2_ and S_Cu3_.

### Biocompatibility-cytotoxicity of films on L292 cells

Determination of the potential cytotoxic effect of various thin film extracts against our in vitro biological system of L929 cells was performed through the Alamar blue assay. Initially, two sets of extracts were prepared from each tested sample, through their immersion into 6-well culture plates containing DMEM media for 72 h and 168 h (details described in Sect. 2.4). Next, extracts obtained from each of the above sets were used in order to investigate their cytotoxic properties against L929 cells, following 72 h of exposures. Interestingly, in order to observe the maximum effect of derived extracts of all tested samples in terms of cytotoxicity against L929 cells, undiluted DMEM anion-leached media was used in this series of experiments. Specifically, cells were exposed to four different extracts of both Cu (S_Cu1,_ S_Cu2,_ S_Cu3,_ S_Cu4_) and Ag (S_Ag1,_ S_Ag2,_ S_Ag3,_ S_Ag4_) doped Ti_3_Au thin films. In addition, extracts prepared from a blank Cu substrate of similar size and under similar conditions, as well as 10% of Dimethyl Sulfoxide (DMSO), were used as positive controls, whereas DMEM (Blank) growth medium along with a blank Ti substrate were used as a negative controls, against L929 mouse fibroblasts.

**Fig. 14:**
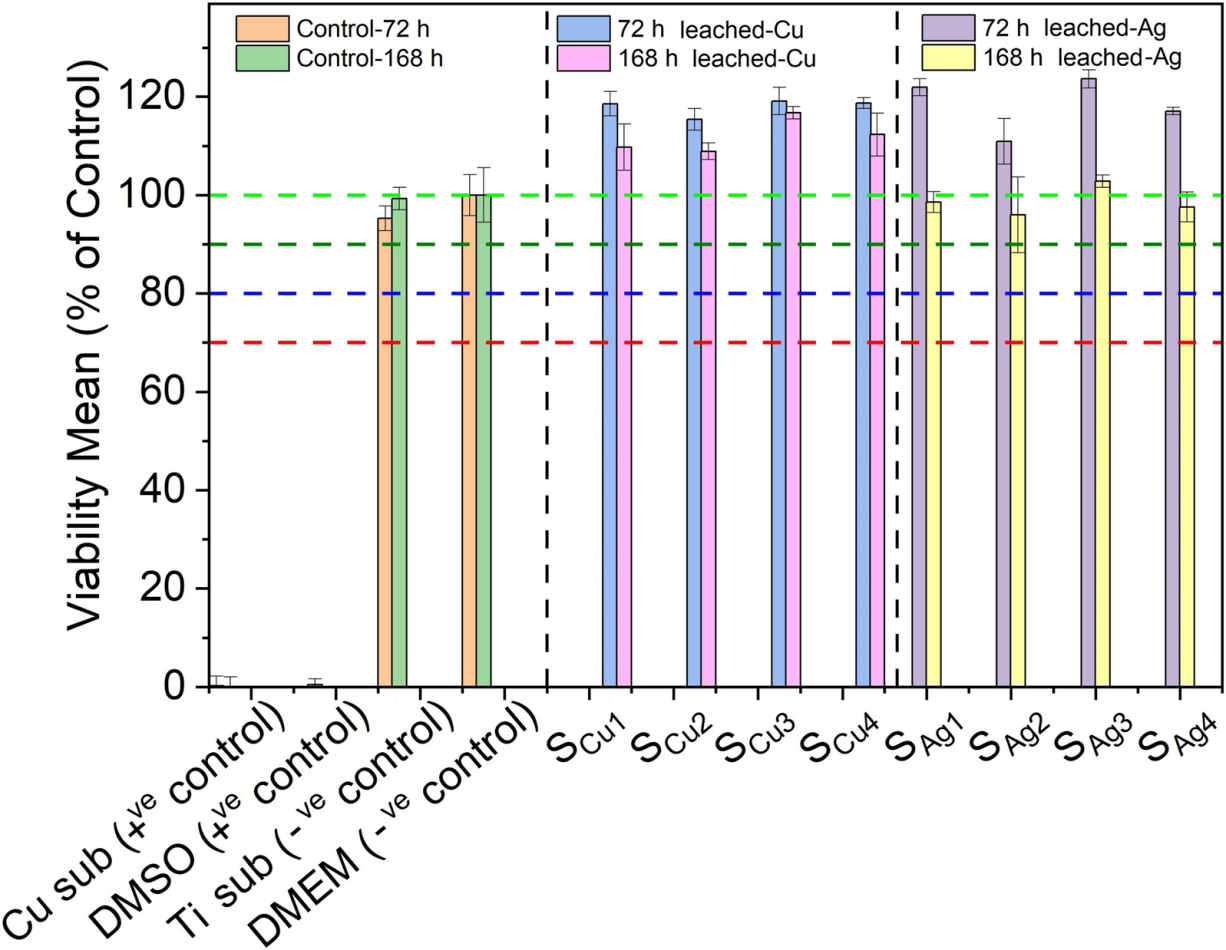
Cytotoxic effect of Ti_3_Au thin films extracts from samples deposited on Ti substrates with varying Cu and Ag doping concentrations, against L929 mouse fibroblast cells, following 72 h of exposure with 72 h and 168 h leached extracts, compared to negative controls (untreated [BLANK] and Ti substrate) and positive controls (Cu substrate and 10% DMSO)

Results following the Alamar blue cytotoxicity assay are presented as bar charts in Fig. 14. Data analysis indicated that both Cu substrate extracts and 10% of DMSO solution exhibited considerable cytotoxic activity, as levels of viable cells were dramatically reduced, opposed to the effect of DMEM (blank sample) and Ti substrate extracts. Specifically, as expected, viability levels of DMEM-treated cells remained unaffected, a pattern also observed in exposures with Ti derived extracts, with viability levels reaching values between 95–100%, even after exposures with extracts of prolonged (168 h) leaching period. Accordingly, all tested Ti_3_Au thin films doped with Cu performed notable biocompatibility rates as were shown to further increase/stimulate L929 viability levels (above 100%) following incubations with leached extracts obtained following 72 h of extraction. Moreover, although levels of viable cells were slightly reduced in exposures with 168 h leached extracts (compared to those of 72 h), were still above 100%, indicating a safe cytotoxic profile. Such slight decline of cell viability rates could be attributed to the increased concentration/release of metal ions in extracts obtained following prolonged leaching duration. In parallel, a similar safe cytotoxic profile was observed when L929 fibroblasts were exposed to all tested Ti_3_Au thin film samples doped with Ag, as once again were capable of stimulating cell proliferation reaching levels above 100% in exposures with 72 h leached samples. Also in this case, although exposures with prolonged (168 h) leaching period derived extracts caused a minor decrease of viability levels, they were still above 90%, suggesting a non-cytotoxic effect and high biocompatible properties.

**Fig. 15:**
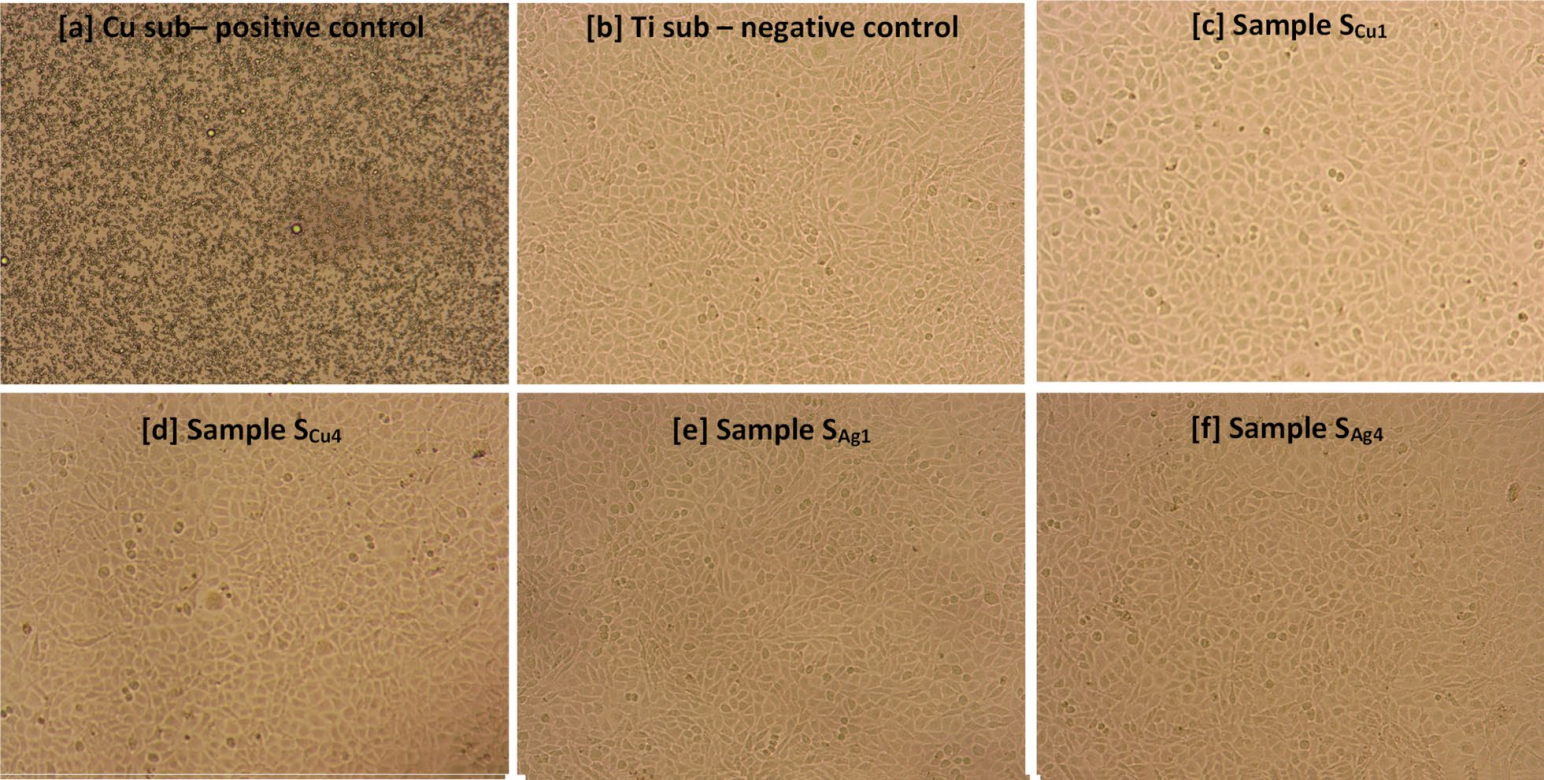
Morphological changes of L929 mouse fibroblast cells following 72 h of incubation with **a** Cu substrate (positive control), **b** Ti substrate (negative control) **c** S_Cu1_, **d** S_Cu4_, **e** S_Ag1_ and **f** S_Ag4_ Ti_3_Au thin film extracts, obtained after 168 h of ion leaching. Images acquired using an inverted Kern microscope with attached digital camera and 10X lens

The above results are further supported by microscopic observation of L929 mouse fibroblast cell’s morphology, following exposures to the sample surfaces for a total of 168 h (Fig. 15). In particular, it can be clearly seen that exposure to the Cu substrate was associated with high rates of cytotoxicity, leading to significant cell shrinkage and death of L929 mouse fibroblast cells, ultimately resulting in confluency reduction (Fig. 15a). On the contrary, cell morphology as well as confluency of L929 cells exposed to Ti (Fig. 15b) and Ag/Cu doped Ti_3_Au thin film extracts (Fig. 15c-f) remained unaffected, indicating a safe cytotoxic profile.

The leached ion concentration measured by performing ICPOEMS analysis on 72 and 168 h leached sample extracts helps to better understand the biocompatible nature of the Ag/Cu doped Ti_3_Au thin films. For the Cu doped samples, no ions could be detected after 72 h of leaching, other than Au ions, which were less than 0.10 ppm. However, when the extraction duration is extended to 168 h, the Cu doped samples begin to show presence of other ion species: Ti ion concentration increases to 0.01–0.02 ppm, whereas Al becomes the dominant species with an ion concentration of 0.17–0.20 ppm. Samples S_cu1_ and S_cu2_ report Cu ion concentrations of 0.07 ppm, which increases to 0.08 ppm for the Cu rich samples S_cu3_ and S_cu4_. On the other hand, Ag doped samples begin to show presence of antimicrobial Ag ions between 0.14–0.15 ppm, even after 72 h of extraction time. There is a further increase of 0.03–0.05 ppm in Ag ion concentration after 168 h of extraction, taking the total leached Ag ion concentration to between 0.17–0.20 ppm. Al ions are another prevalent species in the Ag doped samples, with observed ion concentrations of 0.16–0.19 ppm. Leached ion concentrations of Ti and Au never exceed 0.07 ppm in the Ag doped samples, even after 168 h of extraction, bearing a close resemblance to the Cu doped samples. Various studies have proven that only Ag and Cu ion concentrations exceeding 1 ppm can affect the cell viability of L929 mouse fibroblast cells [73–75], which is in good agreement with the results presented for the Ag/Cu doped Ti_3_Au thin films in this work.

**Fig. 16:**
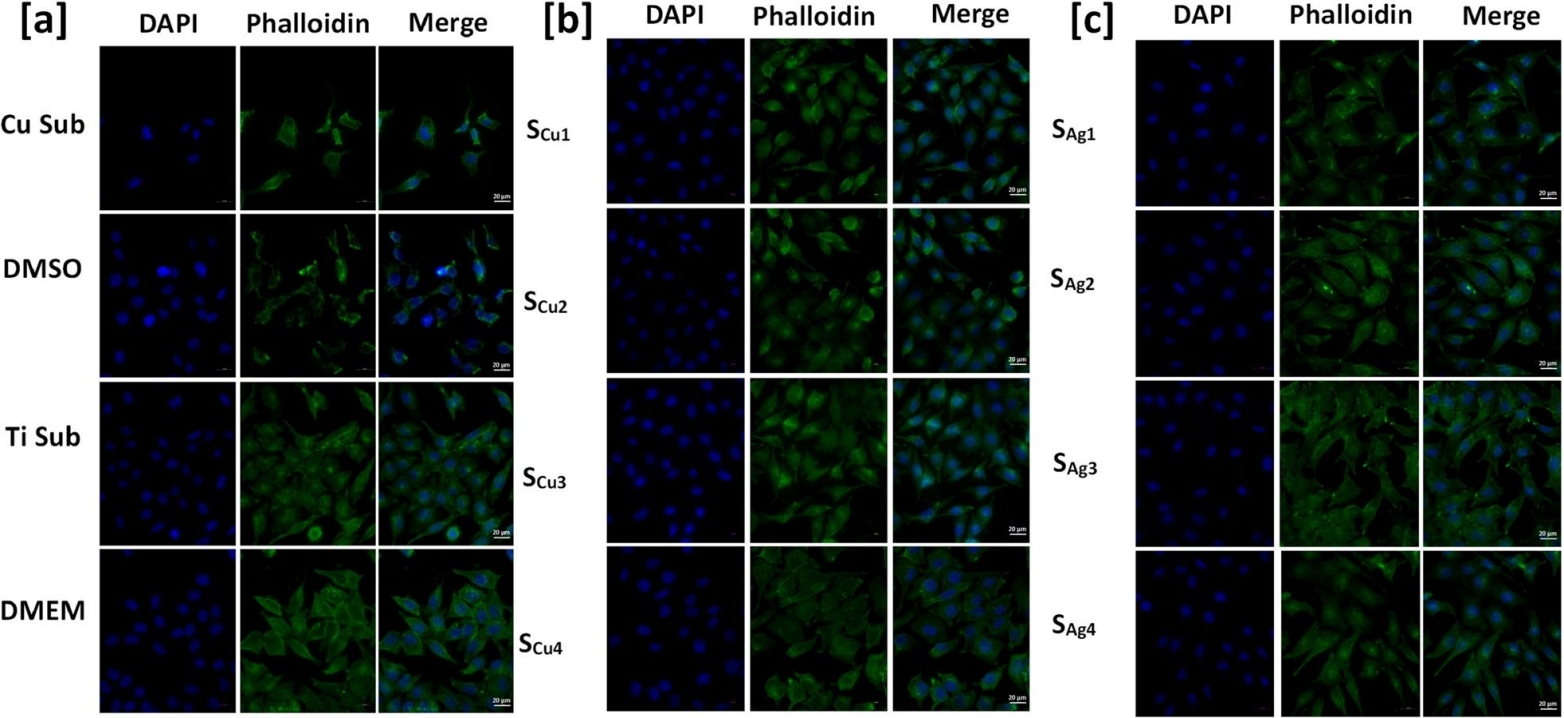
Immunofluorescence images showing the effect of direct exposure to **a** Control samples **b** Cu doped and **c** Ag doped Ti_3_Au thin films on L929 cells’ morphology and actin cytoskeleton. For each sample following exposures, L929 cells were fixed and stained with anti-Phalloidin (middle panel) and counterstained for DNA with DAPI (left panel). Green and blue colour represents staining for Phalloidin and DAPI, respectively, while merged images for Phalloidin/DAPI staining are also shown (right panel). Scale: 20 μm

The effect of direct exposure of Ag and Cu doped Ti_3_Au thin film surfaces on the cell morphology (actin filaments and nuclear DNA) of L929 mouse fibroblasts was observed through fluorescence microscopy using Phalloidin and DAPI staining, which stains actin filaments and nucleus, respectively and the images are presented in Fig. 16 a-c. Along with the thin film test samples, the mouse fibroblast cells were directly incubated with Cu and Ti substrates, used as positive and negative controls, respectively. Further, the exposures to 10% DMSO and DMEM (control) culture media were used as absolute positive and negative cytotoxic controls. Our immunofluorescence results revealed that both actin filaments and nucleus of mouse fibroblasts were not altered following Cu and Ag doped Ti_3_Au thin films exposures (Fig 16b and c), as they appeared intact well-defined and stained, indicating healthy L929 cells, further supporting the biocompatible profile of all tested thin films. This pattern was comparable to that of both DMEM (control) and Ti substrate treated cells (Fig. 16a) showing a normal nucleus staining and well-defined actin filament regions. On the contrary, Cu and 10% DMSO exposures caused significant changes in L929 cells’ morphology, observed as a loss of membrane integrity and an abnormal cell shape and actin filaments staining, indicating a significant cytotoxic effect. Overall, these results clearly show that the Ti_3_Au based coatings can maintain a positive environment for growth and proliferation of cells in biological medium.

**Fig. 17:**
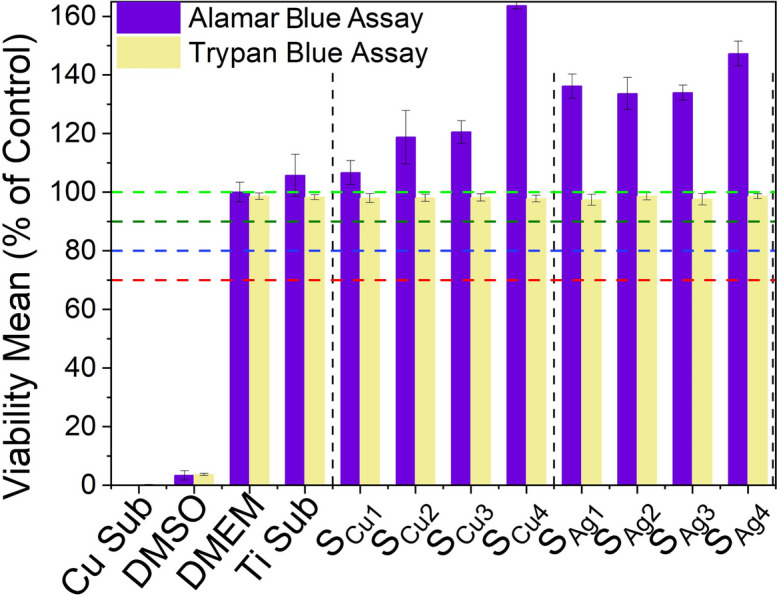
Cytotoxic effect of Ti_3_Au thin films deposited on Ti substrates with varying Cu and Ag doping concentrations, against L929 mouse fibroblast cells, following 72 h of direct exposure to the sample surface, compared to negative controls (untreated [BLANK] and Ti substrate) and positive controls (Cu substrate and 10% DMSO), determined by Trypan blue exclusion staining and Alamar blue assay

The results of cell viability analysis using the Trypan blue exclusion assay are presented in Fig. 17 and show the cell viability levels of L929 mouse fibroblast cells after direct exposure to Ag/Cu dopedTi_3_Au thin film surfaces, measured on the same day as the immunofluorescence analysis for a direct correlation. The results show that the exposures of L929 cells to various Ag and Cu doped thin films does not alter their viability levels, as they are comparable to those of untreated (DMEM/blank) samples. On the other hand, the Cu substrate and 10% of DMSO caused a dramatic reduction in viability of these cells, exhibiting a strong cytotoxic activity. This result is in very good agreement with extract exposure method (Fig. 14) and shows that L929 mouse fibroblast cells remain viable not only in the biological medium containing leached extracts from Ag/Cu doped Ti_3_Au thin film coatings, but also maintain an excellent viability performance when exposed directly on the surface of these thin film coatings.

Furthermore, in order to make a direct comparison with the extract exposure method (Fig 14), viability levels of L929 cells directly exposed to Ag and Cu doped Ti_3_Au thin film surfaces were also studied the next day using the Alamar blue assay as previously described in section 2.4.2, see Figure 17. Results show that both Ag and Cu doped Ti_3_Au thin film coatings present a very safe cytotoxic profile against L929 cells, with very high viability levels. This indicates that Ti_3_Au based thin films possess biocompatibility properties in direct and indirect extract exposure routes, which is a highly suitable trait for coating of artificial joint implants. The L929 cells treated to bare Ti alloy substrate experienced a similar cytotoxicity profile as Ag/Cu doped Ti_3_Au coatings, although to a lesser extent when compared to doped samples, but still above 100%. In contrast, treatment with 10% DMSO resulted in a very strong cytotoxic activity, dramatically decreasing viability levels of exposed L929 cells.

**Fig. 18:**
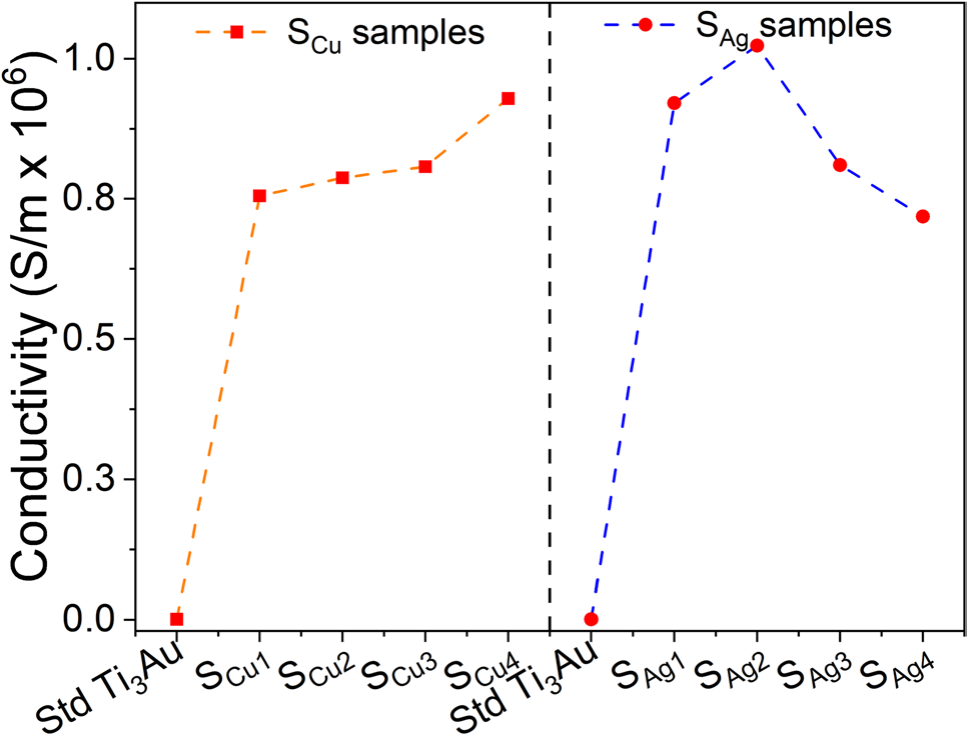
Variation in electrical conductivity of Ti_3_Au thin films samples with variation in Ag/ Cu dopant concentration in the film

Several studies have shown that electrical conductivity of the substrates within a biological system can enhance bioactivity and promote cell response in terms of proliferation and differentiation [76–79]. It has been proposed that electrically conductive medium could encourage electrical communication between myoblasts stimulating increased myogenic differentiations [80]. The results of electrical conductivity for Ag and Cu doped Ti_3_Au thin films measured by a 4-point probe surface measurement system are presented in Fig. 18. For comparison a standard Cu substrate and a 250 nm thick Ag film were also measured with electrical conductivities of 1.84 × 10^5^ and 1.44 × 10^8^ S/m, respectively.

The electrical conductivity of the standard Ti_3_Au thin film is relatively low, having a value of 7.6 × 10^2^ S/m. However, when these samples are doped with Ag or Cu the electrical conductivity increases and is expected because of the high conductivity of Ag and Cu compared to Ti [81–83]. Correlating this result with the theory that electrical conductivity aids cellular proliferation and differentiation, the higher conductivity for Ag/Cu doped samples compared to the standard Ti_3_Au thin film could explain the slight improvement in cell viability performance observed by these samples. The conductivity of Cu doped samples increases at a steady rate with increase in Cu concentration from 0.75 × 10^6^ to 0.92 × 10^6^ S/m. On the other hand, for Ag doped samples, the electrical conductivity initially increases from 0.92 × 10^6^ to 1.02 × 10^6^ with increase in Ag concentration (S_Ag1_ < S_Ag2_) but thereafter falls steadily to 0.81 × 10^6^ and 0.71 × 10^6^ with further increase in Ag content (S_Ag2_ > S_Ag3_ > S_Ag4_). Higher electrical conductivity at lower Ag concentration could be associated to the presence of smaller conductive Ag particles spread more uniformly throughout the Ti_3_Au matrix, as seen in the SEM images for samples S_Ag1_ and S_Ag2_. But as the concentration of Ag grows within the film, Ag particles begin to agglomerate into fewer large island like structures with increased spacing, thereby reducing the electrical conductivity [84–86]. Therefore, by correlating the ICPOEMS and surface conductivity results to the excellent cell viability reported for the Ag/Cu doped samples (> 100%), it can be argued that leached Ag/Cu ion concentrations of 0.07 to 0.20 ppm are not enough to cause any significant cytotoxic effect, however, increases in electrical conductivity caused by addition of these elements could be favourable to stimulate myogenic differentiations.

### Antimicrobial results

**Fig. 19:**
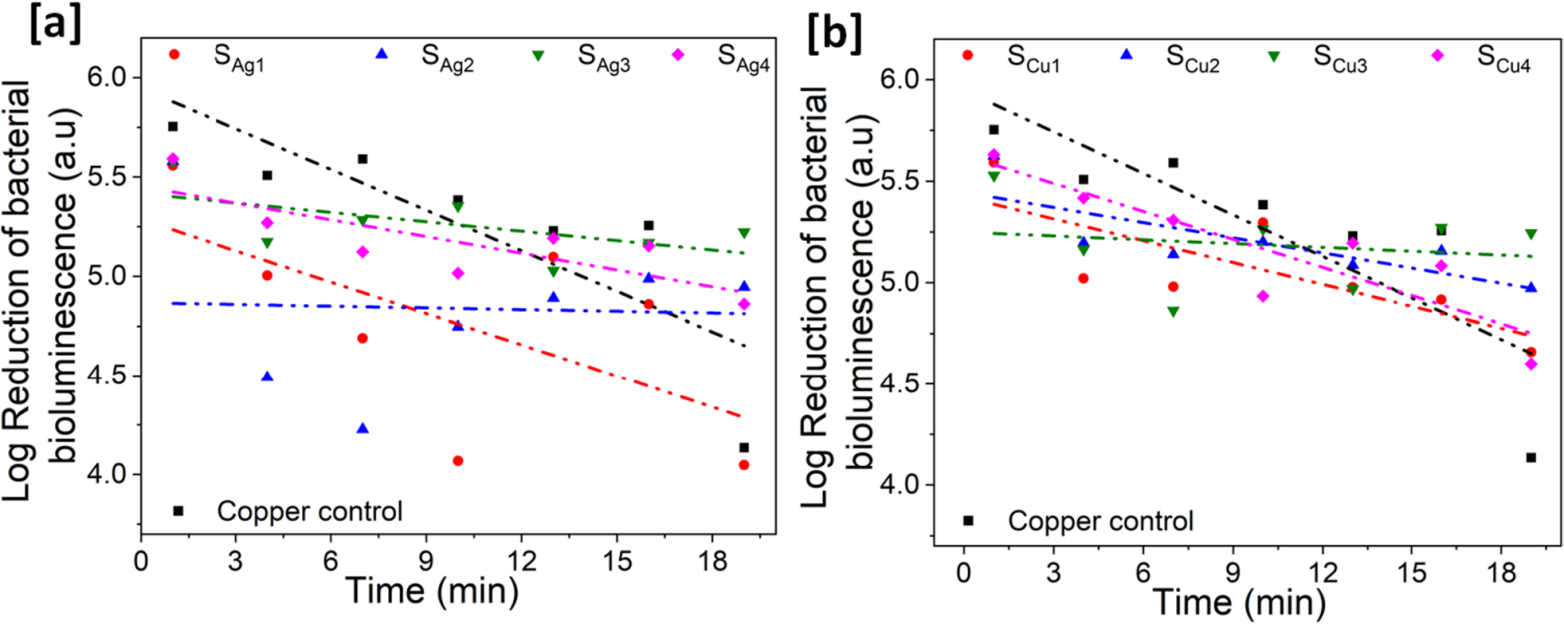
Graphs showing linear fitted trendlines for log reduction of bioluminescence response from E.coli. bacterial colonies under exposure to **a** Ag doped and **b** Cu doped Ti_3_Au thin films

We used the bioluminescence output of *E. coli* pQE-ilux as an index of death. Our in vitro experiments have shown that the loss of bioluminescence correlates very well with bacterial death when challenged with copper ions (data not shown). We consider the assay superior to traditional recovery assays as these equate a reduction in recovery of live cells with death but do not account for recovery artefact relating to differential surface adhesion by the bacteria. Logarithmic reduction in the number of live *E. coli* bacteria emitting bioluminescence after exposure to the sample surfaces was measured in a dark box and used as a reference to indicate the antimicrobial property of the Ag and Cu doped Ti_3_Au thin films, see Fig. 19.

Each data point represents the logarithmic value of averaged bioluminescence intensity emitted by the microbial cells when deposited on the surface of the Ag/Cu doped Ti_3_Au thin films. The spread of data points is fitted using a linear fit of y = mx + c, where x and c represent the slope and y-axis intercept of the line respectively, while the R^2^ value is presented to indicate the quality of data fit. The negative and steeper slope indicates a steeper reduction in the number of *E. coli* colonies and the higher R^2^ value indicates the consistency of this trend across the exposure period. The table for individual slope and R^2^ value for these samples are presented in the Supplementary data 3. The results are compared against a Cu substrate, a known antimicrobial agent and this control sample exhibits a strong antimicrobial property with a 1.6 log reduction in bioluminescence intensity over the test period. For Ag doped Ti_3_Au thin films, sample S_Ag1_ shows the best antimicrobial property, similar to Cu substrate with a 1.6 log reduction over the test duration. The reduction in this sample is steep in the initial 10 min before settling down in the second half of the test period. With further increase in the Ag concentration, the antimicrobial performance diminishes to < 0.8 log reductions for samples S_Ag2_, S_Ag3_ and S_Ag4_ respectively. For the Cu doped samples, sample S_Cu4_ and S_Cu1_ provide the strongest antimicrobial performance of > 1 log reductions, with both samples showing a steady reduction in bacterial bioluminescence. While samples S_Cu2_ and S_Cu3_ only achieve a < 0.5 log reduction over the test period.

## Discussion

There is growing interest in biocompatible thin film coatings with enhanced wear resistance and antimicrobial properties that can be used to coat load-bearing implants to extend their lifetime and help to combat implant associated infections. One thin film material that is receiving increasing attention is β-Ti_3_Au due to its excellent biocompatibility and increased hardness when compared to pure Ti and Ti_6_Al_4_V alloy [1–5]. In this work, we have made the first fundamental attempt to dope the biocompatible β-Ti_3_Au thin film structure with small quantities (< 10 at%) of biocidal Ag and Cu elements to impart strong antimicrobial functionality without affecting the excellent mechanical hardness of this alloy system.

X-ray diffraction patterns revealed that stoichiometric addition of Au in the Ti leads to formation of Ti_3_Au intermetallic at the required 3:1 ratio and addition of small doping concentrations of Ag and Cu are below the required level for the formation of Ti-Ag and Ti-Cu intermetallics [44–46], while thermal energy from the film substrate increases the diffusion of these elements into interstitial points as solid solutes. At higher Cu concentrations Cu ions begin to alloy into the Ti_3_Au matrix causing lattice distortions and in plane stress [41] due to the smaller ionic radius of Cu (1.28 Å) when compared to Ti (1.47 Å) [42, 43]. addition of Ag leads to visible changes in the Ti_3_Au thin film diffraction pattern and therefore more significant changes in the structural arrangement.

Microscopic images of surface topology show that small additions of Cu produces well dispersed spherical grains in the Ti_3_Au matrix, which is in good agreement with previous work on addition of Cu in a Ti matrix [49]. These grains develop more angular features with increasing Cu concentration in the thin film. While small additions of Ag in the matrix are shown to retain the more angular grain features of the pure Ti_3_Au thin film [4] but with further Ag addition the grains become flatter and interspersed with increasing Ag agglomerations, forming large Ag particles on the thin film surface as well as within the columnar cross sectional structure of the Ti_3_Au matrix. This finding is in good agreement with previous studies on Ag doped Ti-based alloys [50] and such Ag precipitation can negatively affect thin film density and resulting mechanical hardness and corrosion resistance [52].

Mechanical characterisation revealed that the elastic modulus of the thin film gradually reduced in proportion to increasing number of Ag solute atoms in the Ti_3_Au matrix [44–46] from 136 to 82 GPa, and variation in results increased due to increasing Ag agglomeration on the surface. Ti_3_Au samples doped with Cu report elastic modulus values very close to that of the standard Ti_3_Au thin films and with more consistent values in the range 134 to 146 GPa and could be related to the higher reported Young’s modulus of Cu (128 GPa) compared to Ti (112 GPa) and Ag (73 GPa) [44, 60], grain refinement and constraining of neighbouring grains [62], and the precipitation of the Ti-Cu intermetallic leading to stiffening of the Ti_3_Au thin film matrix with increasing Cu concentration [44, 46, 61]. While one current limitation with these alloys is that their elastic modulus values are still higher than that of natural bone (30 GPa) [87], the observed reduction in the elastic modulus of the standard Ti_3_Au intermetallic when alloyed with yielding elements like Ag could be further explored to enable load bearing implants to efficiently transfer loads to developing bones and reduce the stress shielding effect [88]. Thin film hardness followed a similar trend to that elastic modulus with a steady decrease with increasing Ag content in the Ti_3_Au thin films. Addition of 0.2 at% Ag is shown to increase the peak mechanical hardness of the standard Ti_3_Au thin film from 12.3 GPa to 14.7 GPa but with further additions of Ag in sample the hardness values reduce and variation in results increase due to Ag agglomeration on the surface. It is known that thin film strengthening can be achieved by increasing barriers to dislocation movement, introducing phase difference, increasing solutes or increasing the internal grain/phase/column boundaries [89, 90] and previous studies have shown that at low Ag concentrations the thermal energy supplied by substrate heating leads to Ag diffusion into the main matrix structure, which act like lattice defects to generate micro-strain [81, 91, 92]. This solid solution of Ag into the main Ti_3_Au coating matrix results in grain refinement or grain boundary reduction, leading to an enhanced solid solution hardening effect [93, 94], as seen for the Ti_3_Au thin film containing 0.2 at% Ag. At higher Ag concentrations, diffusion of Ag starts agglomerating across the grain boundaries and reduces the compactness and hardness of the Ti_3_Au thin films [52]. The hardness of the Cu doped Ti_3_Ag thin films is more consistent in the range 11.6 to 13.2 GPa. Again, a very small Cu dopant concentration leads to diffusion of metal inside Ti_3_Au as a solid solute, creating lattice defects like interstitial occupancy or vacancies, leading to micro strain within the film [95]. Therefore, the hardness increment seen at low Cu concentrations can be related to the effect of growth defects and decrease of crystallite size but with further addition of Cu the hardness undergoes a slight reduction before stabilizing for further samples. Lopes et al*.*, experienced a very similar trend in hardness at intermediate Cu doping concentrations because the low eutectoid point of 7 at% Cu [44] causes the Ti-Cu intermetallics to emerge in an amorphous nature with grain sizes much smaller than the dislocation length, causing plastic deformation and thereby reducing the hardness of the film [95]. With further increment in the dopant concentration, Ti-Cu intermetallics begin to crystallise and therefore with coexistence of quasi-crystalline and nanocrystalline intermetallic structures, the neighbouring grains belong to different lattice structures/phases [95]. This leads to strengthening of the atomic bonds along cluster grains thereby improving the resistance to dislocation movement and increasing mechanical hardness [89, 90, 95]. Addition of 0.2 at% Ag is also shown to significantly decrease the corresponding wear rate of the standard Ti_3_Au thin film from 19.62 × 10^–6^ to 3.03 × 10^–6^ mm^3^/Nm. While Ti_3_Au films doped with small quantities of Cu achieved a minimum wear rate of 5.50 × 10^–6^ mm^3^/Nm. This behaviour is typical in thin film coatings doped with soft materials like Ag and Cu and can be related to trade-off between a reduction in frictional coefficient due to development of a self-lubricating surface layer [63–65] and a decrease in surface hardness [67] with increasing Ag/Cu content in the Ti_3_Au thin films.

The Ag and Cu doped Ti3Au thin films were also shown to have excellent corrosion resistance when tested in SBF solution and cytotoxicity tests revealed that the excellent biocompatibility profile of the standard Ti_3_Au thin film can be further enhanced by addition of electrically conductive Ag and Cu particles into the matrix, with L929 viability levels above 100% following both direct contact with thin film surfaces and incubations with 72 h leached extracts. Exposures with prolonged 168 h leached extracts caused a minor decrease of viability levels to ~ 90% for Ag doped Ti_3_Au thin films, which could be attributed to Ag + ions being 6–8 times more cytotoxic towards L929 mouse fibroblast cells than of Cu2 + ions [73]. However, overall, these results indicate that thin film samples of Ti_3_Au intermetallic maintain the stable non-corrosive and excellent biocompatible profile of the individual Ti and Au elements, even when doped with potential antimicrobial elements like Ag and Cu up to 4.1 and 7.2 at%, respectively.

Antimicrobial test results showed a drastic reduction in microbial survival over a short test period of < 20 min for Ti_3_Au films doped with Ag or Cu. From the Ion leaching results discussed earlier it can be seen that even after 72 h of leaching, the leached Ag ion concentration in the extracts from Ag doped Ti_3_Au thin films do not exceed 0.15 ppm and those of Cu doped Ti_3_Au thin films only record leached Cu ion concentrations of 0.08 ppm. While leached ion concentrations below 1 ppm are favourable for biocompatibility performance of these thin films [73–75], previous studies have shown that Ag and Cu concentrations as low as 2.3–11 ppb are sufficient to initiate antimicrobial activities [30, 35]. Other studies have projected a safe concentration range for Ag and Cu ions where they can be antimicrobial without being cytotoxic as 0.26–0.1 ppm and 0.6–6.3 ppm, respectively [96]. Corelating ion leaching results to antimicrobial performance, it is very promising to see that Ag and Cu doped Ti_3_Au thin films with 80–150 ppb of Ag/Cu ion concentration can produce a visible reduction in bacterial bioluminescence within a short exposure time of < 20 min, where leached ion concentrations would normally be expected to be much lower than those levels after 72 h. Recent studies on kinetics of Ag [97] and Cu [98] ion leaching have explored the effect of exposure time on the leaching potential of these elements and found that with increase in leaching time, initially the leaching rate increases rapidly but then begins to slow until achieving a steady rate. Therefore, the ion release rate in the initial minutes is sufficient to reach the levels required to execute antimicrobial action on *E. coli* colonies. The higher concentration of leached Ag ions from Ag doped samples compared to Cu ions from Cu doped samples, arises from the difference in particle formation behaviour for these two dopants and supports the stronger antimicrobial performance observed for Ag doped samples. These recent studies [97, 98] have also explored the effect of particle size on leaching potential and observed that finer particles accelerate the leaching rate because of larger contact area. The XRD patterns and SEM images clearly show that with increasing dopant concentration, Ag tends to agglomerate to form larger particles protruding from the main Ti_3_Au alloy matrix, whereas Cu is easily dispersed in the matrix, even at the highest doping concentration. Ag agglomeration versus copper dispersion within the Ti_3_Au matrix was also observed to affect the conductivity of these thin films, thereby having a direct effect on the cytotoxicity results. Similarly, the difference in particle formation behaviour explains the better antibacterial performance of Ag doped samples at lower concentration where finer particle size leads to higher ion release during the initial leaching period, compared to Cu doped samples where the best antimicrobial performance was observed at the highest dopant concentration where the Cu particles remained finely dispersed in Ti_3_Au matrix. According to the work of Ning et al. [96], in these concentration ranges, the prevailing mechanism for bacterial death from metal ions is due to formation of reactive oxygenated species (ROS), like the production of hydroxyl free radicals when these ion penetrate the cell membrane. These ROS cause oxidative damage to bacterial cell membranes and proteins, which appears as permeability of the cell membrane and eventually leads to its rupture and loss of cytoplasmic fluid. While this study confirms the antimicrobial performance of Ti_3_Au-Ag/Cu alloy system, and corelates this performance to the release of Ag ions from the implant surface, further in-depth antimicrobial studies are required to establish the exact mechanism behind the antibacterial activity of this material. Overall, the antimicrobial tests strongly support that medical implant surfaces coated with Ag/Cu doped Ti_3_Au thin films can provide enough/sufficient biocidal Ag/Cu ions in the initial minutes after implant surgery to inhibit the growth of infection causing microbial colonies. However, further studies are required in order to explore and characterize the potential of Ag/Cu ions leached from longer exposure durations, extending into hours or even days, on the prevention of biofilm formations protecting bacterial colonies.

## Conclusion

In summary this work has demonstrated that of doping the β-Ti_3_Au thin film structure with small quantities of biocidal Ag and Cu elements can lead to an improvement in the mechanical hardness and wear resistance of the resulting coating without compromising its excellent biocompatibility, whilst also imparting the coating with strong antimicrobial functionality. These findings suggest that β-Ti_3_Au-Ag/Cu thin films could have future potential as a coating material to improve the wear performance and extend the life cycle of medical implant devices and provide sufficient antimicrobial activity to resist biofilm formation during post-surgery healing to reduce the risk of implant associated infections.

### Supplementary Information

**Additional file 1:** **Supplementary data 1.** SEM surface image of sample S_Cu4_.

**Additional file 2:** **Supplementary data 2.** **a** XRD patterns for standard Ti_3_Au thin films developed at varying deposition pressure. **b** Surface SEM images of standard Ti_3_Au thin films **c** Cross section SEM images of standard Ti_3_Au thin films. **d** TEM profile of standard Ti_3_Au thin films. **e** AFM surface profile for standard Ti_3_Au thin film.

**Additional file 3:** **Supplementary data 3.** **a** Table showing the corresponding slope, intercept and R2 values derived from linear fit performed on log reduction of antimicrobial bioluminescence data from Ag and Cu doped samples.

## Data Availability

The datasets used and analyzed during the current study are available from the corresponding author on reasonable request.

## References

[1] Lukose CC (2022). Thermal activation of Ti(1-x)Au(x) thin films with enhanced hardness and biocompatibility. Bioactive Mater.

[2] Svanidze E (2016). High hardness in the biocompatible intermetallic compound β-Ti3Au. Sci Adv.

[3] Xin Y (2019). Microstructure of hard biocompatible Ti1−xAux alloys. Mater Charact.

[4] Karimi A, Cattin C (2018). Ιntermetallic β-Ti3Au hard thin films prepared by magnetron sputtering. Thin Solid Films.

[5] Lukose CC (2023). Adatom controlled emergence of high hardness in biocompatible beta-Ti3Au intermetallic thin film surfaces. Surf Interfaces.

[6] Zaffe D, Bertoldi C, Consolo U (2004). Accumulation of aluminium in lamellar bone after implantation of titanium plates, Ti-6Al-4V screws, hydroxyapatite granules. Biomaterials.

[7] Bocchetta P (2021). Passive Layers and Corrosion Resistance of Biomedical Ti-6Al-4V and β-Ti Alloys. Coatings.

[8] Kumar S, Narayanan TS, Kumar SS (2010). Influence of fluoride ion on the electrochemical behaviour of β-Ti alloy for dental implant application. Corrosion Sci.

[9] Frye BM, Laughery KR, Klein AE (2021). The oxinium arthrogram: a sign of oxidized zirconium implant failure. Arthroplast Today.

[10] Tribe H (2013). Advanced wear of an Oxinium femoral head implant following polyethylene liner dislocation. Ann R Coll Surg Engl.

[11] Zou AH (2019). Liner dissociation leading to catastrophic failure of an Oxinium femoral head. Arthroplast Today.

[12] Kore L (2020). Oxidized zirconium total knee arthroplasty implant failure in a patient with knee instability. Arthroplast Today.

[13] Oh KT (2007). Cytocompatibility and electrochemical properties of Ti-Au alloys for biomedical applications. J Biomed Mater Res B Appl Biomater.

[14] Martinez WE, Gregori G, Mates T (2010). Titanium diffusion in gold thin films. Thin Solid Films.

[15] Rajagopalan M, Gandhi RR (2012). First principles study of structural, electronic, mechanical and thermal properties of A15 intermetallic compounds Ti3X (X=Au, Pt, Ir). Physica B.

[16] Lee Y-R (2014). Effect of gold addition on the microstructure, mechanical properties and corrosion behavior of Ti alloys. Gold Bulletin.

[17] Zaman HA (2015). Metallic biomaterials for medical implant applications: a review. Appl Mech Mater.

[18] Market SM. https://en.wikipedia.org/wiki/Prices_of_chemical_elements#cite_note-smm-titanium-43. February 2020 19 July 2023.

[19] Senkov ON, Miracle DB (2021). Generalization of intrinsic ductile-to-brittle criteria by Pugh and Pettifor for materials with a cubic crystal structure. Sci Rep.

[20] Shahid A (2021). The prospects of antimicrobial coated medical implants. J Appl Biomater Funct Mater.

[21] Pesode PA, Barve SB (2021). Recent advances on the antibacterial coating on titanium implant by micro-Arc oxidation process. Materials Today.

[22] Ishihama H (2021). An antibacterial coated polymer prevents biofilm formation and implant-associated infection. Sci Rep.

[23] Esteves GM (2022). Antimicrobial and antibiofilm coating of dental implants-past and new perspectives. Antibiotics (Basel).

[24] Thukkaram M (2021). Biological activity and antimicrobial property of Cu/a-C: H nanocomposites and nanolayered coatings on titanium substrates. Mater Sci Eng C Mater Biol Appl.

[25] Ponomarev VA (2020). Ag(Pt) nanoparticles-decorated bioactive yet antibacterial Ca- and P-doped TiO2 coatings produced by plasma electrolytic oxidation and ion implantation. Appl Surface Sci.

[26] Foster HA (2011). Photocatalytic disinfection using titanium dioxide: spectrum and mechanism of antimicrobial activity. Appl Microbiol Biotechnol.

[27] Yeung KWK (2013). Antimicrobial effects of oxygen plasma modified medical grade Ti–6Al–4V alloy. Vacuum.

[28] Fu S (2022). A novel Ti-Au alloy with strong antibacterial properties and excellent biocompatibility for biomedical application. Mater Sci Eng C Mater Biol Appl.

[29] Liu X (2016). Antibacterial abilities and biocompatibilities of Ti-Ag alloys with nanotubular coatings. Int J Nanomedicine.

[30] Ewald A (2006). Antimicrobial titanium/silver PVD coatings on titanium. Biomed Eng Online.

[31] Dosunmu E (2015). Silver-coated carbon nanotubes downregulate the expression of Pseudomonas aeruginosa virulence genes: a potential mechanism for their antimicrobial effect. Int J Nanomedicine.

[32] Gordon O (2010). Silver coordination polymers for prevention of implant infection: thiol interaction, impact on respiratory chain enzymes, and hydroxyl radical induction. Antimicrob Agents Chemother.

[33] Ji H (2021). Corrosion and antibacterial performance of novel selective-laser-melted (SLMed) Ti-xCu biomedical alloys. J Alloys Compounds.

[34] Wu JH (2020). Effect of Ti2Cu precipitation on antibacterial property of Ti-5Cu alloy. Mater Sci Eng C Mater Biol Appl.

[35] Javadhesari SM, Alipour S, Akbarpour MR (2020). Biocompatibility, osseointegration, antibacterial and mechanical properties of nanocrystalline Ti-Cu alloy as a new orthopedic material. Colloids Surf B Biointerfaces.

[36] Roy S, Hasan I, Guo B (2023). Recent advances in nanoparticle-mediated antibacterial applications. Coordination Chemist Rev.

[37] Birkett M (2022). Recent advances in metal-based antimicrobial coatings for high-touch surfaces. Int J Mol Sci.

[38] Mu J (2021). Improved wear and corrosion resistance of biological compatible TiZrNb films on biomedical Ti6Al4V substrates by optimizing sputtering power. Surface Coatings Technol.

[39] Le DP (2009). Corrosion characteristics of polyaniline-coated 316L stainless steel in sulphuric acid containing fluoride. Corros Sci.

[40] Gregor C (2018). Strongly enhanced bacterial bioluminescence with the ilux operon for single-cell imaging. Proc Natl Acad Sci U S A.

[41] Ginting M, et al. Preparation and characterization of zinc oxide doped with ferrite and chromium. 2017.

[42] Luo J (2005). Effects of the doping element on crystal structure and magnetic properties of Sm(Co, M)7 compounds (M=Si, Cu, Ti, Zr, and Hf). Intermetallics.

[43] Hu Q (2017). Parametric study of amorphous high-entropy alloys formation from two new perspectives: atomic radius modification and crystalline structure of alloying elements. Sci Rep.

[44] Kikuchi M, Takahashi M, Okuno O (2006). Elastic moduli of cast Ti-Au, Ti-Ag, and Ti-Cu alloys. Dent Mater.

[45] Takahashi M, Kikuchi M, Takada Y (2015). Takada, Mechanical properties of dental Ti-Ag alloys with 22.5, 25, 27.5, and 30 mass% Ag. Dent Mater J.

[46] Takahashi M (2006). Electrochemical behavior of cast ti-ag alloys. Dent Mater J.

[47] Patterson AL (1939). The scherrer formula for X-ray particle size determination. Phys Rev.

[48] Wojcieszak D (2020). Influence of material composition on structure, surface properties and biological activity of nanocrystalline coatings based on Cu and Ti. Coatings.

[49] Stranak V (2014). Ionized vapor deposition of antimicrobial Ti–Cu films with controlled copper release. Thin Solid Films.

[50] Zamponi C, Wuttig M, Quandt E (2007). Ni–Ti–Ag shape memory thin films. Scripta Mater.

[51] Kusano E (2019). Structure-zone modeling of sputter-deposited thin films: a brief review. Appl Sci Converg Technol.

[52] Hong C (2021). Effect of silver content on the microstructure, thermal stability and mechanical properties of CrNx/Ag nanocomposite films. Ceram Int.

[53] Gerberich WW (2006). An energy balance criterion for nanoindentation-induced single and multiple dislocation events. J Appl Mech.

[54] Wang Y, Tam PL, Shen YG (2008). Behavior of Ti0.5Al0.5N thin film in nanoscale deformation with different loading rates. Thin Solid Films.

[55] Corcoran SG (1996). nanoindentation studies of yield point phenomena on gold single crystals. MRS Online Proc Libr.

[56] Ding J, Meng Y, Wen S (2000). Mechanical properties and fracture toughness of multilayer hard coatings using nanoindentation. Thin Solid Films.

[57] Oliver WC, Pharr GM (1992). An improved technique for determining hardness and elastic modulus using load and displacement sensing indentation experiments. J Mater Res.

[58] Chuang CT (2008). Effects of internal stresses on the mechanical properties of deposition thin films. J Mater Process Technol.

[59] Nanda Kumar AK (2006). TEM and nanoindentation studies on sputtered Ti40Ni60 thin films. Mater Chem Phys.

[60] Benjamin DC. American Society for Metals. Handbook, Metals handbook Vol.2, Properties and selection, nonferrous alloys and pure metals. 9th ed.. ed. 1979, Metals Park, Ohio: Metals Park, Ohi: American Society for Metals.

[61] Souza SA (2009). Effect of cooling rate on Ti–Cu eutectoid alloy microstructure. Mater Sci Eng, C.

[62] Callisti M (2013). Effects of Cu on the microstructural and mechanical properties of sputter deposited Ni-Ti thin films. Surf Coat Technol.

[63] Mi P, Ye F (2018). Influence of the Cu addition on the tribological properties of HVOF sprayed bimodal WC-Co coating. Materials Res Express.

[64] Echavarría AM, Bejarano GG, Meza JM (2017). Mechanical and tribological features of TaN(Ag-Cu) duplex nanocomposite coatings: their response to heat treatment. Ingeniare. Ingeniare Revista Chilena De Ingeniería.

[65] Xu X (2022). Microstructure and tribological performance of adaptive MoN–Ag nanocomposite coatings with various Ag contents. Wear.

[66] Zhao Y, Xu J, Peng S (2021). Synthesis and evaluation of TaC nanocrystalline coating with excellent wear resistance, corrosion resistance, and biocompatibility. Ceram Int.

[67] Liu X (2015). The combined effects of Cu and Ag on the nanostructure and mechanical properties of CrCuAgN PVD coatings. Surf Coat Technol.

[68] Bita AI (2022). Electrochemical and in vitro biological evaluation of bio-active coatings deposited by magnetron sputtering onto biocompatible Mg-0.8Ca alloy. Materials (Basel).

[69] Olayinka A, Esther A, Philip O (2021). Examination of electrochemical corrosion properties of titanium carbide thin film grown by RF magnetron sputtering. Materials Today.

[70] Shi K (2019). Electrochemical properties of niobium coating for biomedical application. Coatings.

[71] Li H (2018). Influence of C2H2 Flows on microstructure and corrosion resistance of TiCN films doped with carbon atoms. Coatings.

[72] Ogawa ES (2016). Surface-treated commercially pure titanium for biomedical applications: Electrochemical, structural, mechanical and chemical characterizations. Mater Sci Eng C Mater Biol Appl.

[73] Elshahawy WM, Watanabe I, Kramer P (2009). In vitro cytotoxicity evaluation of elemental ions released from different prosthodontic materials. Dent Mater.

[74] Cao B (2012). Concentration-dependent cytotoxicity of copper ions on mouse fibroblasts in vitro: effects of copper ion release from TCu380A vs TCu220C intra-uterine devices. Biomed Microdevices.

[75] Milheiro A (2016). In vitro cytotoxicity of metallic ions released from dental alloys. Odontology.

[76] Aparicio-Collado JL (2020). Novel semi-interpenetrated polymer networks of poly(3-Hydroxybutyrate-co-3-Hydroxyvalerate)/Poly (Vinyl Alcohol) with incorporated conductive polypyrrole nanoparticles. Polymers (Basel).

[77] Cui L (2020). Electroactive composite scaffold with locally expressed osteoinductive factor for synergistic bone repair upon electrical stimulation. Biomaterials.

[78] Chen J (2018). Conductive nanofibrous composite scaffolds based on in-situ formed polyaniline nanoparticle and polylactide for bone regeneration. J Colloid Interface Sci.

[79] Jun I, Jeong S, Shin H (2009). The stimulation of myoblast differentiation by electrically conductive sub-micron fibers. Biomaterials.

[80] Aparicio-Collado JL (2022). Electroactive calcium-alginate/polycaprolactone/reduced graphene oxide nanohybrid hydrogels for skeletal muscle tissue engineering. Colloids Surf B Biointerfaces.

[81] Lopes C (2015). Evolution of the functional properties of titanium–silver thin films for biomedical applications: Influence of in-vacuum annealing. Surf Coat Technol.

[82] Lopes C (2015). Study of the electrical behavior of nanostructured Ti–Ag thin films, prepared by glancing angle deposition. Mater Lett.

[83] Lopes C (2015). Multifunctional Ti–Me (Me=Al, Cu) thin film systems for biomedical sensing devices. Vacuum.

[84] Leng J, et al. Influence of Ag thickness on structural, optical, and electrical properties of ZnS/Ag/ZnS multilayers prepared by ion beam assisted deposition. J Appl Phys. 2010;108(7).

[85] Shen M (2021). Effects of AgTi3 intermetallic on suppression of Ag agglomeration: a theoretical study. Mol Simul.

[86] Jang J, Choi J-W (2021). Silver alloy-based metal matrix composites: a potential material for reliable transparent thin film heaters. J Materials Chemistry C.

[87] Morgan EF, Unnikrisnan GU, Hussein AI (2018). Bone mechanical properties in healthy and diseased States. Annu Rev Biomed Eng.

[88] Li Y (2014). New developments of Ti-based alloys for biomedical applications. Materials (Basel).

[89] Mayrhofer PH (2006). Microstructural design of hard coatings. Prog Mater Sci.

[90] Hultman L (2000). Thermal stability of nitride thin films. Vacuum.

[91] Vaz F (2004). Structural, optical and mechanical properties of coloured TiNxOy thin films. Thin Solid Films.

[92] Cunha L (2006). Structural evolution in ZrNxOy thin films as a function of temperature. Surf Coat Technol.

[93] Vieira MT (1999). The influence of silver on the structure and mechanical properties of (TiAl)-based intermetallics. Thin Solid Films.

[94] Li WZ (2013). Influence of Al content on the mechanical properties and thermal stability in protective and oxidation atmospheres of Zr–Cr–Al–N coatings. Surf Coat Technol.

[95] Lopes C (2020). Evolution of the mechanical properties of Ti-based intermetallic thin films doped with different metals to be used as biomedical devices. Appl Surface Sci.

[96] Ning C (2015). Concentration ranges of antibacterial cations for showing the highest antibacterial efficacy but the least cytotoxicity against mammalian cells: implications for a new antibacterial mechanism. Chem Res Toxicol.

[97] Al-Zubeidi A (2021). Single-particle hyperspectral imaging reveals kinetics of silver ion leaching from alloy nanoparticles. ACS Nano.

[98] Shi S-X (2022). Kinetic characteristics and mechanism of copper leaching from waste printed circuit boards by environmental friendly leaching system. Process Saf Environ Prot.

