# Supplementary figures and images for: Biocompatible Ti3Au–Ag/Cu thin film coatings with enhanced mechanical and antimicrobial functionality
Source: Biomater Res. 2023 Sep 25;27:93. doi: 10.1186/s40824-023-00435-1 (PMC10521510; doi:10.1186/s40824-023-00435-1)

Supplementary data 1:


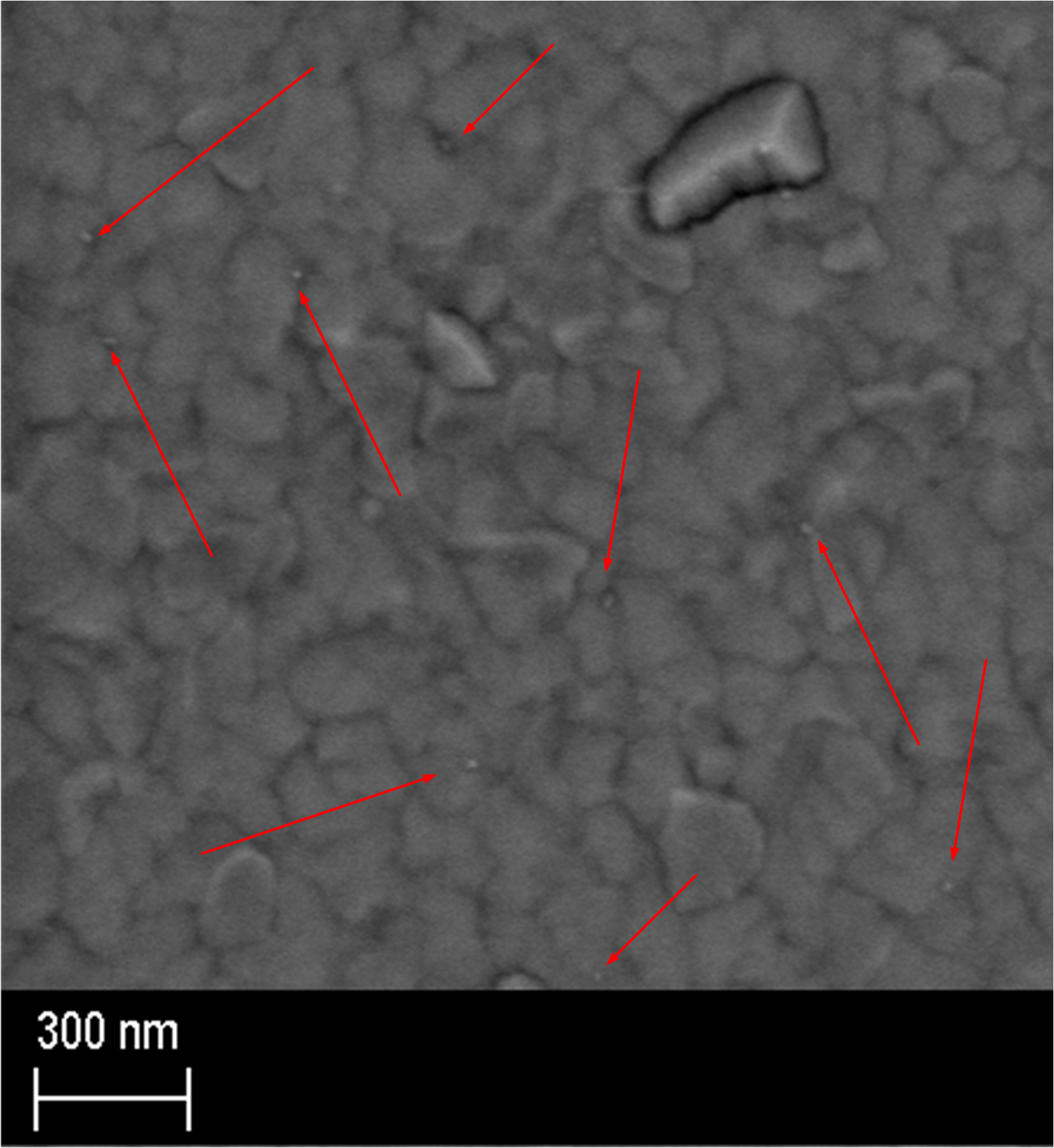


**Supplementary data 1:** SEM surface image of sample SCu4.

Supplement: Supplementary file 1 — Additional file 1: Supplementary data 1. SEM surface image of sample SCu4. [file 40824_2023_435_MOESM1_ESM.docx]
