# Supplementary material for: Biocompatible Ti3Au–Ag/Cu thin film coatings with enhanced mechanical and antimicrobial functionality
Source: Biomater Res. 2023 Sep 25;27:93. doi: 10.1186/s40824-023-00435-1 (PMC10521510; doi:10.1186/s40824-023-00435-1)
Supplement: Supplementary file 2 — Additional file 2: Supplementary data 2. a XRD patterns for standard Ti3Au thin films developed at varying deposition pressure. b Surface SEM images of standard Ti3Au thin films c Cross section SEM images of standard Ti3Au thin films. d TEM profile of standard Ti3Au thin films. e AFM surface profile for standard Ti3Au thin film. [file 40824_2023_435_MOESM2_ESM.docx]

Supplementary data 2:


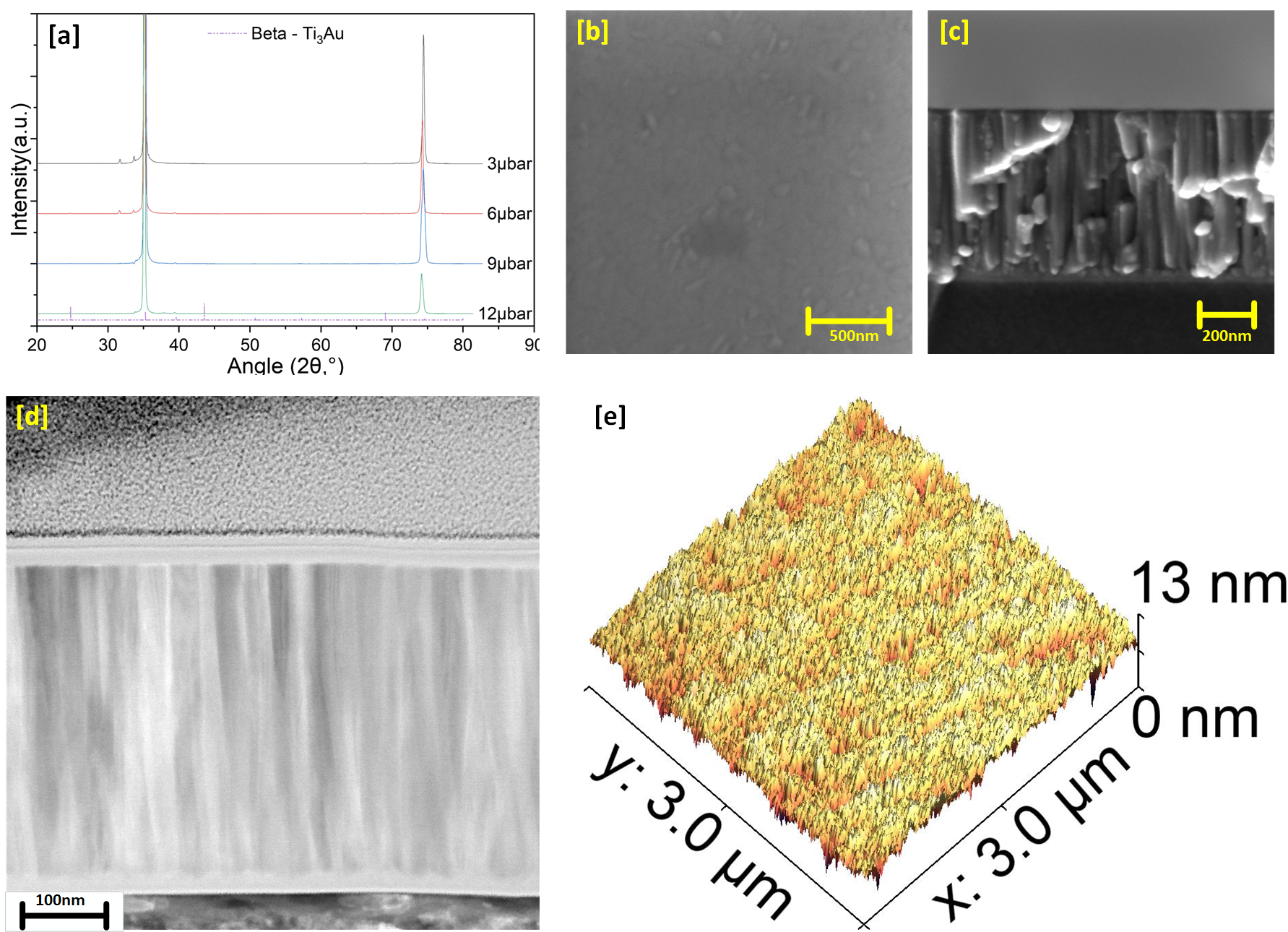


**Supplementary data 1:** (a) XRD patterns for standard Ti_3_Au thin films developed at varying deposition pressure. (b) Surface SEM images of standard Ti_3_Au thin films (c) Cross section SEM images of standard Ti_3_Au thin films. (d) TEM profile of standard Ti_3_Au thin films. (e) AFM surface profile for standard Ti_3_Au thin film.
