# Supplementary material for: Biocompatible Ti3Au–Ag/Cu thin film coatings with enhanced mechanical and antimicrobial functionality
Source: Biomater Res. 2023 Sep 25;27:93. doi: 10.1186/s40824-023-00435-1 (PMC10521510; doi:10.1186/s40824-023-00435-1)
Supplement: Supplementary file 3 — Additional file 3: Supplementary data 3. a Table showing the corresponding slope, intercept and R2 values derived from linear fit performed on log reduction of antimicrobial bioluminescence data from Ag and Cu doped samples. [file 40824_2023_435_MOESM3_ESM.docx]

Supplementary data 3:

| **Sample type** | **Sample name** | **Slope value** | **Intercept** | **R^2^** |
| --- | --- | --- | --- | --- |
| Control | Glass | - 0.02447 | 6.19081 | 0.80428 |
|  | Copper control | - 0.06812 | 5.94666 | 0.68912 |
|  | Ti control | - 0.04644 | 5.46135 | 0.61489 |
| Ag doped Ti_3_Au thin films | S_Ag1_ | - 0.05255 | 5.28636 | 0.38428 |
|  | S_Ag2_ | - 0.00287 | 4.86709 | 0.00191 |
|  | S_Ag3_ | - 0.01581 | 5.41699 | 0.35011 |
|  | S_Ag4_ | - 0.02807 | 5.45308 | 0.64067 |
| Cu doped Ti_3_Au thin films | S_Cu1_ | - 0.03588 | 5.42211 | 0.60564 |
|  | S_Cu2_ | - 0.02487 | 5.44503 | 0.62226 |
|  | S_Cu3_ | - 0.00627 | 5.24905 | 0.03467 |
|  | S_Cu4_ | - 0.04603 | 5.62736 | 0.78658 |

**Supplementary data 3:** (a) Table showing the corresponding slope, intercept and R2 values derived from linear fit performed on log reduction of antimicrobial bioluminescence data from Ag and Cu doped samples.
